# Metformin exerts multitarget antileukemia activity in JAK2^V617F^-positive myeloproliferative neoplasms

**DOI:** 10.1038/s41419-017-0256-4

**Published:** 2018-02-22

**Authors:** João Agostinho Machado-Neto, Bruna Alves Fenerich, Renata Scopim-Ribeiro, Christopher A. Eide, Juan Luiz Coelho-Silva, Carlos Roberto Porto Dechandt, Jaqueline Cristina Fernandes, Ana Paula Nunes Rodrigues Alves, Priscila Santos Scheucher, Belinda Pinto Simões, Luciane Carla Alberici, Lorena Lôbo de Figueiredo Pontes, Cristina E. Tognon, Brian J. Druker, Eduardo Magalhães Rego, Fabiola Traina

**Affiliations:** 1 Department of Internal Medicine, University of São Paulo at Ribeirão Preto Medical School, Ribeirão Preto, São Paulo, Brazil; 2 Knight Cancer Institute, Oregon Health & Science University, Portland, OR USA; 3 Howard Hughes Medical Institute, Portland, OR USA; 4 Department of Physics and Chemistry, Faculty of Pharmaceutical Sciences of Ribeirão Preto, University of São Paulo, Ribeirão Preto, Brazil; 5 Present Address: Department of Pharmacology, Institute of Biomedical Sciences of the University of São Paulo, São Paulo, Brazil

## Abstract

The recurrent gain-of-function JAK2^V617F^ mutation confers growth factor-independent proliferation for hematopoietic cells and is a major contributor to the pathogenesis of myeloproliferative neoplasms (MPN). The lack of complete response in most patients treated with the JAK1/2 inhibitor ruxolitinib indicates the need for identifying novel therapeutic strategies. Metformin is a biguanide that exerts selective antineoplastic activity in hematological malignancies. In the present study, we investigate and compare effects of metformin and ruxolitinib alone and in combination on cell signaling and cellular functions in JAK2^V617F^-positive cells. In JAK2^V617F^-expressing cell lines, metformin treatment significantly reduced cell viability, cell proliferation, clonogenicity, and cellular oxygen consumption and delayed cell cycle progression. Metformin reduced cyclin D1 expression and RB, STAT3, STAT5, ERK1/2 and p70S6K phosphorylation. Metformin plus ruxolitinib demonstrated more intense reduction of cell viability and induction of apoptosis compared to monotherapy. Notably, metformin reduced Ba/F3 JAK2^V617F^ tumor burden and splenomegaly in Jak2^V617F^ knock-in-induced MPN mice and spontaneous erythroid colony formation in primary cells from polycythemia vera patients. In conclusion, metformin exerts multitarget antileukemia activity in MPN: downregulation of JAK2/STAT signaling and mitochondrial activity. Our exploratory study establishes novel molecular mechanisms of metformin and ruxolitinib action and provides insights for development of alternative/complementary therapeutic strategies for MPN.

## Introduction

Philadelphia chromosome-negative myeloproliferative neoplasms (MPN), including essential thrombocythemia (ET), polycythemia vera (PV) and primary myelofibrosis (PMF), are characterized by excessive myeloid proliferation and have heightened risk for acute myeloid leukemia (AML) transformation^1^. Constitutive activation of the JAK2/STAT signaling pathway is a hallmark of these diseases and plays an important role for MPN pathogenesis. Ruxolitinib is a selective JAK1/2 inhibitor approved by the FDA for the treatment of intermediate and high-risk PMF, and PV patients with inadequate response or intolerant to hydroxyurea. In PMF patients, ruxolitinib is well tolerated, reduces inflammatory cytokines and splenomegaly, and ameliorates constitutional symptoms^2–4^. In PV patients, ruxolitinib controls the hematocrit, reduces the spleen volume, and improves symptoms^5^. However, ruxolitinib treatment does not reverse bone marrow fibrosis and does not lead to elimination of the malignant clone, suggesting the need for new therapeutic approaches to further improve patient responses.

Metformin (1,1-dimethylbiguanide) is a biguanide widely prescribed for the treatment of type II diabetes and metabolic syndromes. In recent years, studies using cancer cell lines and murine models have provided evidence for potential anticancer activity of metformin^6,7^. Some molecular mechanisms for this activity have been proposed, including inhibition of energetic metabolism, cell proliferation and survival signaling pathways, which may occur in an AMPK-dependent or AMPK-independent manner^8–10^. In addition, preclinical studies testing the combination of chemotherapeutic agents with metformin have appeared promising in the treatment of some solid tumors^11^.

Considering that metformin has been proposed to be selective for hematological malignant cells^12–16^ and that metformin has been used for a long time for the treatment of metabolic diseases, preclinical studies to assess the effect metformin may be interesting in MPN, since these findings have potential for incorporation in clinical practice. In the present study, we investigate the cellular and molecular effects of treatment with metformin alone and in combination with ruxolitinib in JAK2^V617F^ MPN models.

## Results

### Metformin reduces cell viability, proliferation, clonogenicity and cell cycle progression in HEL and SET2 cells

**Fig. 1:**
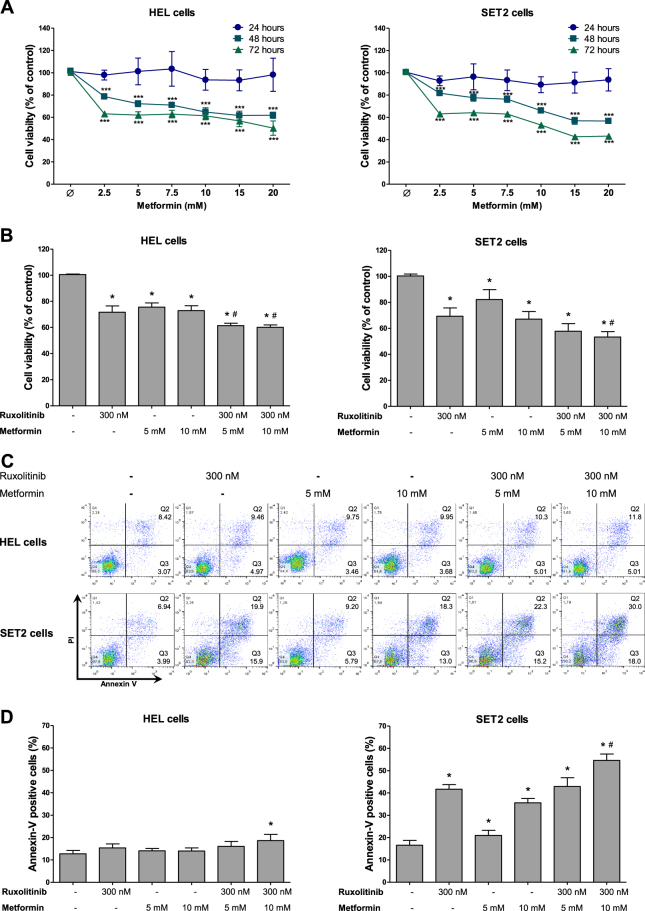
Metformin potentiates ruxolitinib-induced cell viability reduction in JAK2^V617F^ cells. **a** Dose-response and time-response cytotoxicity curves analyzed by methylthiazoletetrazolium (MTT) assay for HEL and SET2 cells treated with metformin for 24, 48 and 72 h. Values are expressed as the percentage of viable cells for each condition relative to untreated controls. Results are shown as the mean ± SD of four independent experiments. ***p < 0.0001 for metformin-treated cells vs. untreated cells; ANOVA test and Bonferroni post-test, all pairs were analyzed and statistically significant differences are indicated. **b** Cell viability was determined by MTT assay in HEL or SET2 cells treated, or not, with the indicated concentrations of ruxolitinib and/or metformin for 48 h and normalized to corresponding untreated cells. Bar graphs represent the mean ± SD of at least four independent experiments. **c** Apoptosis was detected by flow cytometry in HEL or SET2 cells treated with ruxolitinib and/or metformin for 48 h using an annexin V/PI staining method. Representative dot plots are shown for each condition; the upper and lower right quadrants (Q2 plus Q3) cumulatively contain the apoptotic population (annexin V+ cells). **d** Bar graphs represent the mean ± SD of at least four independent experiments quantifying apoptotic cell death. The *p* values and cell lines are indicated in the graphs. *p < 0.05 for metformin-treated and/or ruxolitinib-treated cells vs. untreated cells, #*p* < 0.05 for metformin-treated or ruxolitinib-treated cells vs. combination treatment at the corresponding doses; ANOVA test and Bonferroni post-test, all pairs were analyzed and statistically significant differences are indicated

To characterize the potential efficacy of metformin in human JAK2^V617F^-positive cells, we first investigated the effects of metformin treatment on cell viability in HEL and SET2 cells. In both JAK2^V617F^ cell lines analyzed, metformin reduced cell viability in a dose-dependent and time-dependent manner (Fig. 1a). The IC_50_ values for metformin in HEL and SET2 cells were 18 and 10 mM at 72 h, respectively. Based on previous studies using leukemia cell lines^17^ and our IC_50_ results, we decided to use metformin at 5 and/or 10 mM for in vitro studies. Next, we evaluated the effects of metformin alone and in combination with ruxolitinib on JAK2^V617F^ cell lines by methylthiazoletetrazolium (MTT) assay. In HEL and SET2 cells, treatment with either ruxolitinib or metformin alone significantly reduced the cell viability (*p* < 0.05), and the combination of ruxolitinib (300 nM) plus metformin (10 mM) significantly decreased cell viability when compared with monotherapy (*p* < 0.05) (Fig. 1b). Synergy analysis indicates that the combination of ruxolitinib ≥300 nM with metformin presented synergistic effects in HEL cells (all combination index (CI) values <0.60). In SET2 cells, combinations of ruxolitinib ≤300 nM or metformin ≤15 mM varied from slight synergism (CI: 0.85–0.90), moderate synergism (CI: 0.70–0.85), to synergism (CI < 0.60); the combinations of ruxolitinib >300 mM or metformin >15 mM showed no synergistic effects (Supplementary Figure 1).

**Fig. 2:**
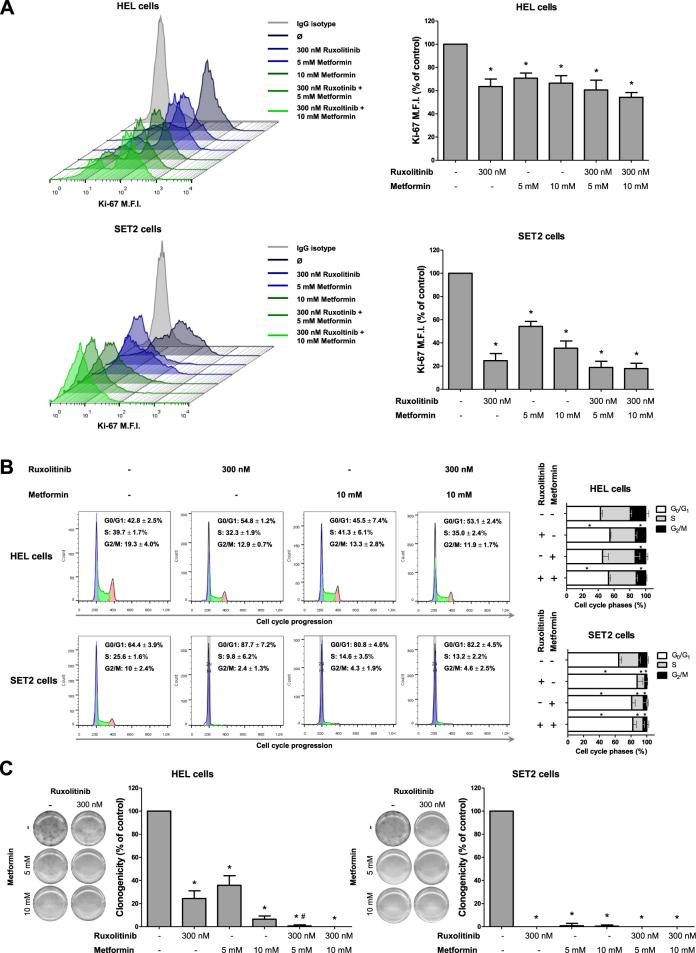
Metformin and ruxolitinib reduce cell proliferation and delay cell cycle progression in HEL and SET2 cells. **a** Ki-67 mean fluorescence intensity (MFI) was determined by flow cytometry after incubation of HEL or SET2 cells treated with ruxolitinib and/or metformin for 48 h; histogram traces are illustrated. The bar graphs represent the Ki-67 M.F.I normalized to the respective untreated control cells, and results are shown as mean ± SD of four independent experiments; *p < 0.05, ANOVA test and Bonferroni post-test, all pairs were analyzed and statistically significant differences are indicated. **b** Cell cycle progression was determined by BD Cycletest™ Plus DNA Reagent Kit in HEL or SET2 cells treated with the indicated concentrations of ruxolitinib and/or metformin for 48 h. A representative histogram for each condition is illustrated. Bar graphs represent the mean ± SD of the percent of cells in G_0_/G_1_, S and G_2_/M phase upon ruxolitinib (300 nM) and/or metformin (10 mM) for 48 h and represent at least four independent experiments. The *p* values and cell lines are indicated in the graphs. *p < 0.05 for metformin-treated and/or ruxolitinib-treated cells vs. untreated cells; ANOVA test and Bonferroni post-test, all pairs were analyzed and statistically significant differences are indicated. **c** Colonies containing viable cells were detected by MTT after 10 days of culture of HEL and SET2 cells treated with ruxolitinib and/or metformin and normalized to the corresponding untreated controls. Colony images are shown for one experiment and the bar graphs show the mean ± SD of at least four independent experiments. The *p* values and cell lines are indicated in the graphs: *p < 0.05 for metformin-treated and/or ruxolitinib-treated cells vs. untreated cells, #*p* < 0.05 for metformin-treated or ruxolitinib-treated cells vs. combination treatment at the corresponding doses; ANOVA test and Bonferroni post-test, all pairs were analyzed and statistically significant differences are indicated

Given these findings, the effects of metformin combined with ruxolitinib were further assayed with respect to cell death and proliferation. Similar to findings for cell viability, SET2 cells subjected to metformin (5 and 10 mM) or ruxolitinib (300 mM) treatment significantly induced apoptosis, and combined treatment (10 mM metformin plus 300 nM ruxolitinib) presented higher levels of apoptosis compared with monotherapy (*p* < 0.05). By contrast, in HEL cells only the combination of metformin (10 mM) plus ruxolitinib (300 nM) significantly induced apoptosis (Fig. 1c,d). Ki-67 analysis revealed that treatment with metformin or ruxolitinib alone or in combination significantly reduced cell proliferation, but no additive effect of the combination was observed in HEL and SET2 cells (Fig. 2a).

In both JAK2^V617F^ cell lines, metformin and/or ruxolitinib resulted in a delay of cell cycle progression, with significant reduction in the percentage of cells in G_2_/M phase (Fig. 2b); long-term exposure to metformin or ruxolitinib also strongly decreased clonogenicity. Interestingly, combined treatment (10 mM metformin plus 300 nM ruxolitinib) completely inhibited colony formation (Fig. 2c).

### Metformin modulates the JAK2/STAT signaling pathway in HEL and SET2 cells

**Fig. 3:**
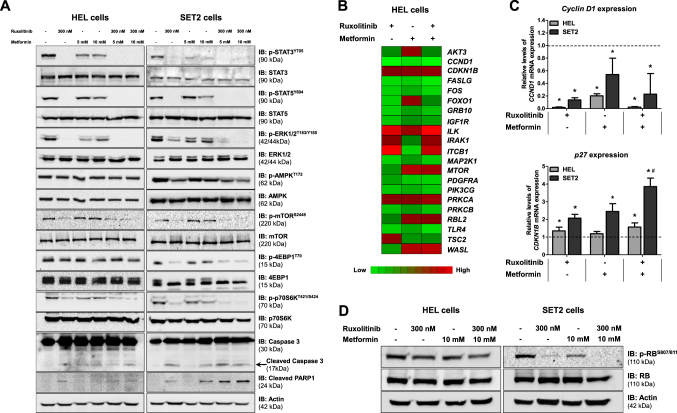
Metformin and ruxolitinib modulate JAK2/STAT signaling and PI3K/AKT-related genes in HEL and SET2 cells. **a** Western blot analysis for p-STAT3^Y705^, p-STAT5^Y694^, p-ERK1/2^T183/Y185^, p-AMPK^T172^, p-mTOR^S2448^, p-4EBP1^T70^, p-p70S6K^T421/S424^, caspase 3 (total and cleaved) and cleaved PARP1 levels in total cell extracts from HEL and SET2 cells treated with the indicated concentrations of ruxolitinib and/or metformin; membranes were reprobed with the antibody for the detection of the respective total protein or actin, and developed with the SuperSignal™ West Dura Extended Duration Substrate system using a Gel Doc XR+ imaging system. **b** Gene expression heatmap from qPCR array analysis of HEL cells treated with ruxolitinib (300 nM) and/or metformin (10 mM). mRNA levels are normalized to those of untreated HEL cells and calculated as fold change in expression; genes demonstrating ≥1.5-fold in either direction compared to untreated cells in any treatment are included in the heat map. Two independent experiments of each condition were used for the analysis; green indicates repressed mRNA levels and red elevated mRNA levels. **c** qPCR analysis of *CCND1* and *CDKN1B* mRNA expression in HEL and SET2 cells treated with ruxolitinib (300 nM) and/or metformin (10 mM) for 48 h. The dashed line represents the mean gene expression in untreated cells and bars represent the fold change in gene expression in HEL and SET2 cells treated with ruxolitinib, metformin, or both compared to their respective untreated cells. The *p* values and cell lines are indicated in the graphs. *p < 0.05 for metformin-treated and/or ruxolitinib-treated cells vs. untreated cells, #*p* < 0.05 for metformin-treated or ruxolitinib-treated cells vs. combination treatment at the corresponding doses; ANOVA test and Bonferroni post-test, all pairs were analyzed and statistically significant differences are indicated. **d** Western blot analysis for p-RB^S807/811^levels in total cell extracts from HEL and SET2 cells treated with ruxolitinib and/or metformin; membranes were reprobed with the antibody for the detection of the total RB protein and actin

We next sought to compare the effects of metformin and ruxolitinib on activation of the JAK2/STAT and mTOR/p70S6K/4EBP1 signaling pathways. As expected, western blot analysis revealed that ruxolitinib (300 nM) was able to reduce STAT3, STAT5, ERK1/2, mTOR, 4EBP1 and p70S6K phosphorylation in HEL and SET2 cells. Surprisingly, treatment with metformin (5 and 10 mM) neither increased AMPK activation nor decreased mTOR phosphorylation in HEL and SET2 cells. Metformin reduced activation of STAT3, STAT5, ERK1/2, 4EBP1 and p70S6K, albeit to a lesser extent than ruxolitinib (Fig. 3a and Supplementary Figure 2). High levels of cleaved caspase 3 and cleaved PARP1 were observed following treatments with metformin and ruxolitinib alone and in combination in SET2 cells (Fig. 3a and Supplementary Figure 2).

### Metformin and ruxolitinib modulate PI3K/AKT-related genes in HEL and SET2 cells

Next, using PCR array, we investigated the effects of metformin alone and in combination with ruxolitinib on expression of PI3K/AKT-related genes in HEL cells. A total of 21 different genes demonstrated a change in expression of ≥1.5-fold in either direction compared to untreated cells: five downregulated genes (*CCND1*, *FASLG*, *ITGB1*, *PDGFRA* and *TLR4*) and six upregulated genes (*AKT3*, *FOXO1*, *ILK*, *MTOR*, *RBL2* and *WASL*) by metformin treatment alone; 11 downregulated genes (*CCND1*, *FASLG*, *FOS*, *FOXO1*, *GRB10*, *IGF1R*, *MAP2K1*, *PDGFRA*, *PIK3CG*, *PRKCB* and *TLR4*) and three upregulated genes (*ILK*, *ITGB1* and *TSC2*) by ruxolitinib treatment alone; and ten downregulated genes (*CCND1*, *FASLG*, *FOS*, *GRB10*, *IGF1R*, *MAP2K1*, *PDGFRA*, *PIK3CG*, *PRKCB* and *TLR4*) and seven upregulated genes (*CDKN1B*, *ILK*, *IRAK1*, *ITGB1*, *MTOR*, *PRKCA* and *WASL*) by the combination (Fig. 3b; Supplementary Table 1). Based on our findings indicating a cytostatic effect of metformin, we choose two genes (*CCND1* and *CDKN1B*) involved in cell proliferation to validate in a larger number of experiments using HEL and SET2 cells, by qPCR. Metformin and ruxolitinib treatment alone or in combination significantly reduced *CCND1* expression (*p* < 0.05) in both JAK2^V617F^ cell lines. Ruxolitinib alone or in combination with metformin increased *CDKN1B* expression in HEL cells. In contrast, treatment with either metformin or ruxolitinib alone increased *CDKN1B* expression in SET2 cells, and this effect was enhanced by combined treatment (*p* < 0.05; Fig. 3c). In conjunction with these findings, we observed that phosphorylation of RB, a cyclin D1 target and a key cell cycle progression-related protein^18^, was reduced following metformin, ruxolitinib, or combination treatment in both JAK2^V617F^ cell lines, though more prominently in SET2 cells (Fig. 3d).

### Metformin reduces cell viability and potentiates ruxolitinib effects in JAK2^V617F^-driven cellular and allograft models

**Fig. 4:**
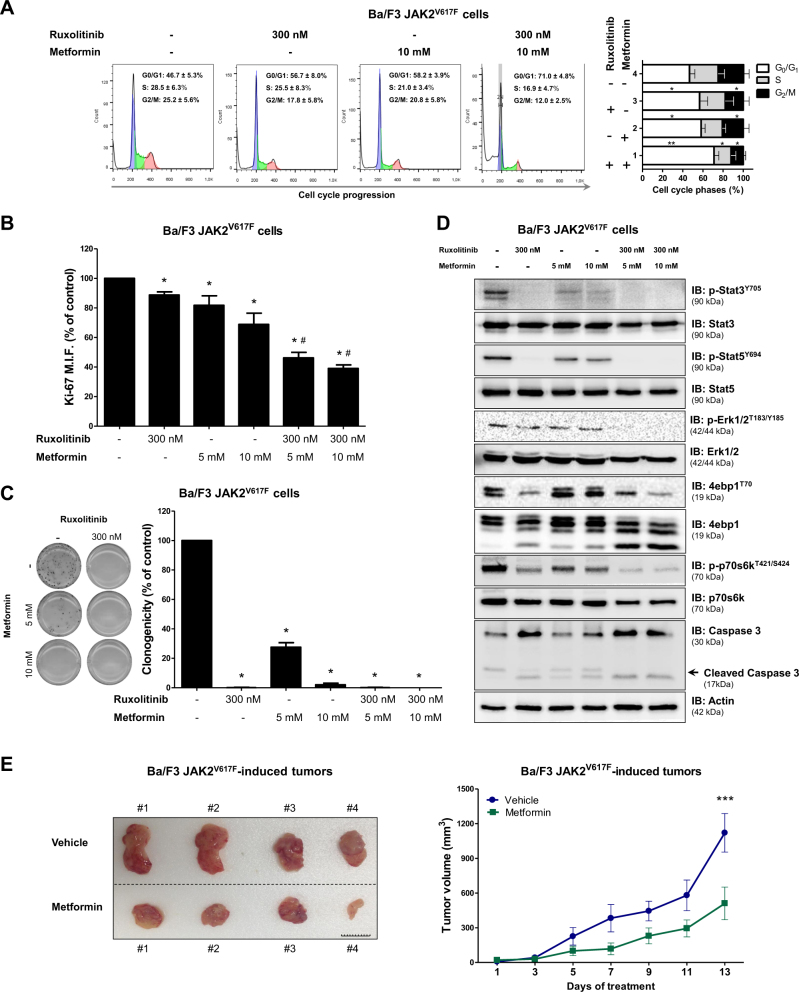
Metformin delays cell cycle progression, reduces colony formation, downregulates JAK2/STAT activation and decreases tumor burden in Ba/F3 JAK2^V617F^ cells. **a** Cell cycle phase profiling was determined by BD Cycletest™ Plus DNA Reagent Kit in Ba/F3 JAK2^V617F^ cells treated with ruxolitinib and/or metformin for 24 h. A representative histogram for each condition is illustrated. Bar graphs represent the mean ± SD of the fraction of cells in G_0_/G_1_, S and G_2_/M phase for each treatment condition across at least four independent experiments. **b** Ki-67 MFI was determined by flow cytometry after incubation of Ba/F3 JAK2^V617F^ cells treated with the indicated concentrations of ruxolitinib and/or metformin for 24 h. The Ki-67 M.F.I was normalized to the respective untreated control cells and results are shown as the mean ± SD of four independent experiments. **c** Colonies containing viable cells were detected by MTT after 10 days of culture of Ba/F3 JAK2^V617F^ cells treated with ruxolitinib and/or metformin and normalized to the corresponding untreated controls. Colony images are shown for one experiment and the bar graphs show the mean ± SD of at least four independent experiments. **d** Western blot analysis for p-Stat3^Y705^, p-Stat5^Y694^, p-Erk1/2^T183/Y185^, p-4ebp1^T70^, p-p70s6k^T421/S424^ and caspase 3 (total and cleaved) levels in total cell extracts from Ba/F3 JAK2^V617F^ cells treated with ruxolitinib and/or metformin for 24 h; membranes were reprobed with the antibody for the detection of the respective total protein or actin, and developed with the SuperSignal™ West Dura Extended Duration Substrate system and a Gel Doc XR+ system. **e** Images and volumes (mean ± SEM) of tumors induced by subcutaneous injection of Ba/F3 JAK2^V617F^ cells in NSG mice, treated with vehicle (PBS) (*n* = 4) or metformin (125 mg/kg/day) (*n* = 4). Tumor volume (V) was calculated using the formula (*V* = *W*^2^ × *L* × 0.52), where *W* and *L* represent the smallest and largest diameters, respectively. Images of individual animal tumors are shown; Scale Bar: 10 mm. The *p* values and cell lines are indicated in the graphs. *p < 0.05, **p < 0.01, ***p < 0.001 for metformin-treated and/or ruxolitinib-treated cells vs. untreated-cells, #*p* < 0.05 for metformin-treated or ruxolitinib-treated cells vs. combination treatment at the corresponding doses; ANOVA test and Bonferroni post-test, all pairs were analyzed and statistically significant differences are indicated

Ba/F3 cells are an IL3-dependent line which, when expressing the *JAK2*^V617F^ mutant gene, acquire IL3-independent growth^19^. Using Ba/F3 JAK2^WT^ and Ba/F3 JAK2^V617F^ models, we evaluated the effect of metformin and/or ruxolitinib on cell viability and apoptosis in the presence or absence of IL-3-rich Wehi-3B-conditioned medium. With or without supplemented Wehi-3B conditioned medium, both metformin and ruxolitinib were able to reduce cell viability and induce apoptosis to similar levels in both Ba/F3 JAK2^WT^ and Ba/F3 JAK2^V617F^ cells, and combined treatment presented more prominent efficacy (*p* < 0.05; Supplementary Figure 3). Based on these results, we performed subsequent experiments using only IL3-deprived Ba/F3 JAK2^V617F^ cells, which are a model where cell viability is supported by the JAK2^V617F^ mutant’s oncogenic potential. Treatment with metformin, ruxolitinib, or the combination induced cell cycle arrest, reduced cell proliferation and clonogenicity, and downregulated STAT3, STAT5 and p70S6K phosphorylation (Fig. 4a–d). Metformin treatment (125 mg/kg/day) was able to reduce the tumor burden of Ba/F3 JAK2^V617F^ cells implanted into NSG mice (*p* < 0.05; Fig. 4e and Supplementary Figure 4).

### Metformin reduces the oxygen consumption in JAK2^V617F^ cells

**Fig. 5:**
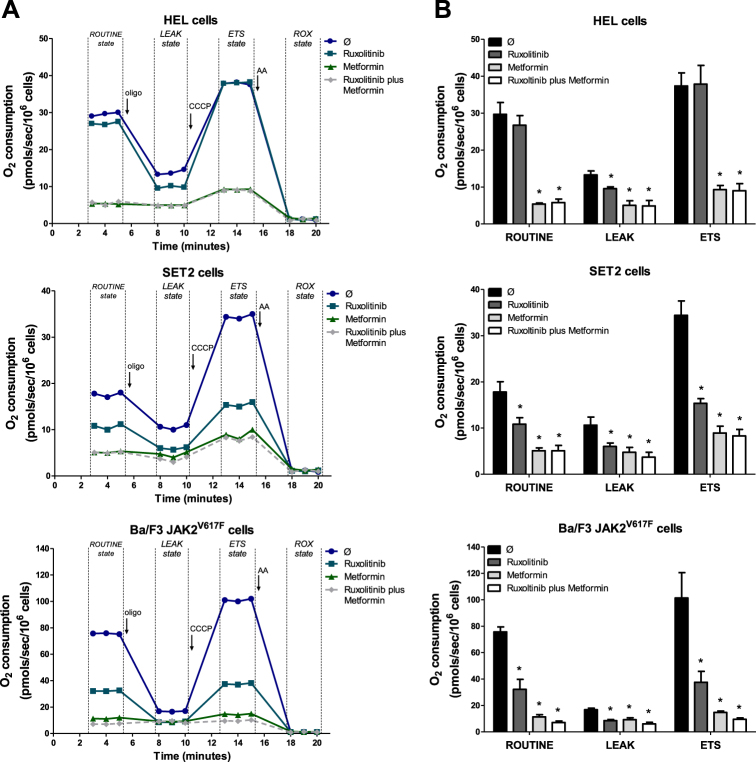
Metformin reduces the oxygen consumption of JAK2^V617F^ cells. **a** Oxygen consumption was determined in HEL, SET2 or Ba/F3 JAK2^V617F^ cells following treatment with ruxolitinib (300 nM) and/or metformin (10 mM) for 24 h using a high-resolution respirometry. A representative line graph containing oxygen consumption at ROUTINE, LEAK, ETS and ROX states is illustrated. The black arrows indicate the sequential addition of oligomycin (oligo, 1 mg/mL), protonophore carbonyl cyanide m-chlorophenyl hydrazone (CCCP, 2 μM) and antimycin A (AA, 3 μM); oxygen consumption rates were measured over time. **b** Bar graphs represent the mean ± SD rate of oxygen consumption mean at ROUTINE, LEAK and ETS states of at least six independent experiments. Values of respiratory rates at ROX state were subtracted from the other states. The *p* values and cell lines are indicated in the graphs. *p < 0.05 for metformin-treated and/or ruxolitinib-treated cells vs. untreated cells; ANOVA test and Bonferroni post-test, all pairs were analyzed and statistically significant differences are indicated

Metformin has been described as a mitochondrial complex I inhibitor in cancer cells^13,20^. Thus, we investigated the impact of metformin alone or in combination with ruxolitinib treatment on oxygen consumption in JAK2^V617F^-positive cell lines using a high-resolution respiratory assay. We observed a strong reduction in cellular oxygen consumption at the state supported by exogenous substrates in culture media (ROUTINE state), at the non-phosphorylating state (LEAK State) and at the maximum respiratory capacity (ETS state) in metformin-treated and metformin plus ruxolitinib-treated HEL, SET2 and Ba/F3 JAK2^V617F^ cells (*p* < 0.05). Unexpectedly, ruxolitinib also significantly reduced oxygen consumption as a single agent at ROUTINE, LEAK and ETS states in SET2 and Ba/F3 JAK2^V617F^ cells, and at LEAK state in HEL cells (Fig. 5).

### Metformin reduces splenomegaly in a Jak2^V617F^ knockin-induced MPN mouse model

**Fig. 6:**
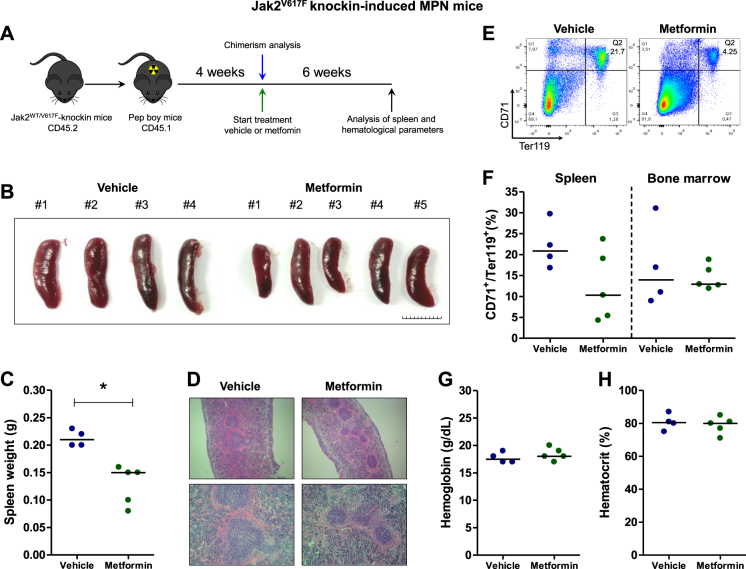
Metformin reduces splenomegaly in Jak2^V617F^ knockin-induced MPN mice. **a** Experimental design for induction of MPN phenotype in mice. Bone marrow cells from Jak2^V617F^ mice were transplanted into lethally irradiated Pep boy mice. After chimerism evaluation at 4 weeks, mice were randomized and daily treated with vehicle (*n* = 4) or metformin (125 mg/kg) (*n* = 5) for 6 weeks. **b** Spleen images and **c** weight of vehicle and metformin-treated MPN mice. Scale Bar: 10 mm.*p < 0.05, Mann–Whitney test. **d** Representative histopathology H&E sections of spleen from vehicle and metformin-treated mice. Magnification of 40× (upper panel) and 100× (lower panel). **e** Illustrative dot plots of erythroid progenitor analysis in the spleen. **f** Dispersion graphs showing the percentage of early erythroid progenitors (CD71^+^/Ter119^+^ cells) in spleen and bone marrow. **g** Dispersion graphs showing the hemoglobin and **h** hematocrit levels

In order to formally assess the effect of metformin treatment using an in vivo model of MPN-like disease, we induced MPN phenotype by transplantation of cells from Jak2^WT/V617F^ knockin mice into Pep boy recipient mice (Fig. 6a). In Jak2^V617F^ knockin-induced MPN animals, metformin treatment (125 mg/kg/day) was well-tolerated, reduced splenomegaly (*p* = 0.02, Fig. 6b,c) and improved splenic architecture (Fig. 6d). Erythroid progenitors in the spleen and bone marrow did not differ between control and metformin-treated groups, but the smaller spleens (from metformin-treated mice #1 and #3) presented a low percentage of erythroid progenitors (4.2 and 5.4%, respectively) (Fig. 6e,f). Hemoglobin and hematocrit were not modulated by metformin treatment (Fig. 6g,h). In Pep boy recipient mice transplanted with Jak2^WT/WT^, metformin treatment (125 mg/kg/day) was well-tolerated and altered neither hematological parameters nor spleen size or architecture (Supplementary Figure 5).

### Metformin inhibits erythropoietin-independent colony formation of primary PV cells

**Fig. 7:**
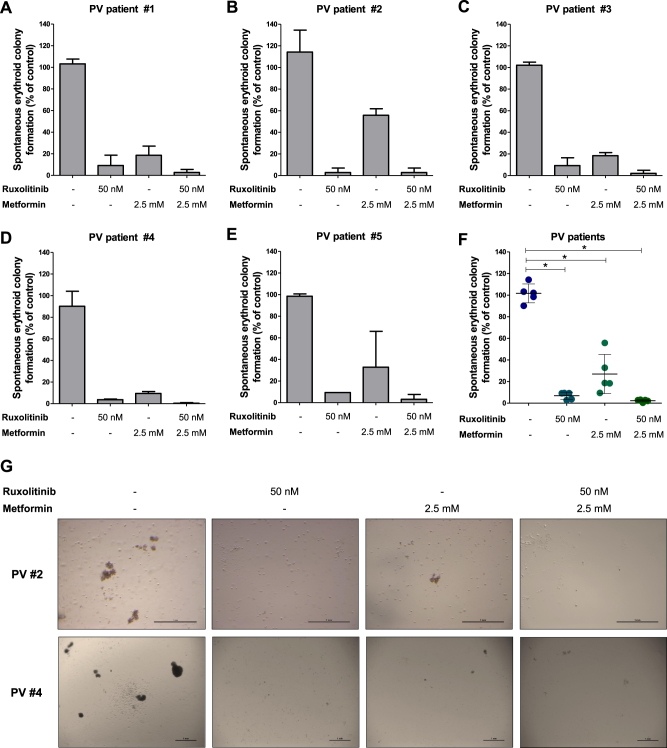
Metformin reduces spontaneous erythroid colony formation in primary polycythemia vera patient cells. **a**–**e** Peripheral blood or bone marrow mononuclear cells from 5 polycythemia vera (PV) patients were plated in methylcellulose, containing cytokines but lacking erythropoietin, in the presence or not of metformin and/or ruxolitinib. Spontaneous erythroid colonies were counted after 14 days of culture and are represented as the percent of untreated controls. Bars indicate the mean ± SD of the duplicate assays for each patient. **f** Dispersion graph comparing combined colony formation results from all five PV patients; the horizontal line represents the mean ± SD. The *p* values are indicated in the graphs; *p < 0.05 for metformin- and/or ruxolitinib-treated cells vs. untreated controls; ANOVA test and Bonferroni post-test, all pairs were analyzed and statistically significant differences are indicated. **g** Representative images of erythropoietin-independent colony formation at 14 days of culture from two PV patients are illustrated

Spontaneous erythroid colony formation in the absence of erythropoietin is a common finding in cells from PV patients cultured in methylcellulose^21,22^. We performed erythropoietin-independent colony assays to verify the effects of metformin (2.5 mM) with and without ruxolitinib (50 nM) in primary cells from PV patients. We observed that metformin was able to reduce spontaneous erythroid colony formation in cells from all PV patients tested. Ruxolitinib presented a strong reduction in erythropoietin-independent colony formation and no additional effects were observed with the combined treatment (Fig. 7).

## Discussion

Herein, we have characterized in vitro and in vivo efficacy of metformin in the context of JAK2^V617F^-driven MPN models. Our results show that metformin reduces cell viability, proliferation, cell cycle progression and clonogenicity in JAK2^V617F^ cells. Metformin is a biguanide used in the treatment of type II diabetes and metabolic syndromes, and epidemiological studies suggest that the drug exerts an antineoplastic activity in humans^23^. In addition, preclinical studies already include a range of evidence indicating a potential therapeutic use for metformin in cancer, and clinical trials are currently in progress (www.clinicaltrials.gov)^6^. In hematological neoplasms, antineoplastic effects of metformin have been described in chronic myeloid leukemia^17,24^, AML^9,12,25,26^, acute lymphoblastic leukemia (ALL)^8,16,24,27,28^, chronic lymphocytic leukemia^29,30^, lymphoma^15^ and multiple myeloma^13^. Importantly, metformin effects appear to act selectively in cancer cells, since cytotoxicity was observed only on leukemia cells and not for colonies from normal hematopoeitic cells^12^, culture of normal peripheral blood mononuclear cells^13,14^, normal cord blood CD34^+^ cells^15^ or normal T lymphocytes^16^.

In the present study, AMPK activation was not modulated by metformin treatment in JAK2^V617F^ cells. Several mechanisms have been proposed to underlie metformin activity and are commonly grouped into (i) AMPK-dependent or (ii) AMPK-independent, both of which have already been described in hematological malignancies. Leclerc and colleagues^8^ observed that metformin induces apoptosis in ALL cell lines, which was abrogated by AMPK silencing, indicating that metformin-induced apoptosis in these cell lines was AMPK-dependent. Scotland and colleagues^9^, using AML cell lines, reported that metformin inhibits cell proliferation and increases apoptosis in an AMPK-independent manner.

Notably, a modest effect was observed with the combination of metformin and ruxolitinib, suggesting that both drugs may share some similarities in mechanisms of action in these cells. In our study, metformin was able to inhibit STAT3 and STAT5 phosphorylation even in the presence of the JAK2^V617F^ mutation in HEL, SET2 and Ba/F3 JAK2^V617F^ cell lines. The inhibitory effect of metformin on STAT3 phosphorylation was reported in breast cancer^31^, esophageal squamous cell carcinoma^32^, lung cancer^33–35^, bladder cancer^36,37^ and pancreatic cancer^38^. There are no reports of metformin effects on STAT5 phosphorylation in solid tumors. Recently, Kawashima and Kirito^39^ showed that metformin reduces cell viability and downregulates the JAK2/STAT5 axis in an AMPK-dependent and PP2A activation-dependent manner in HEL and SET2 cells. In contrast, our results suggest that STAT5 downregulation occurs independently of AMPK activation in JAK2^V617F^ cells.

We also observed downregulation of cyclin D1 (*CCND1*) and upregulation of p27 (*CDKN1B*) by PCR array and qPCR in metformin-treated and/or ruxolitinib-treated HEL and SET2 cells, which may explain the delay in the cell cycle, the reduction of RB phosphorylation and the cytostatic effect, highlighting our findings regarding JAK2/STAT signaling inhibition by metformin. Supporting these results, it is well established that STAT5 activation leads to p27 inhibition, promoting cell cycle progression in HEL cells^40^, and that STAT3 and/or STAT5 activation induces cyclin D1 expression in several types of cells^41,42^. Reduced ERK1/2 activation may also contribute to metformin-induced and/or ruxolitinib-induced cell cycle delay, since the MAPK pathway directly acts on the cell cycle machinery by regulating the expression and/or localization of cyclin D1 and p27^43,44^. The CDK4/6-cyclin D complex phosphorylates RB protein, which represents a critical gatekeeper of cell cycle progression from G_1_ to S phase^18^. In our study, cyclin D1 reduction was consistent with the reduction of RB phosphorylation in JAK2^V617F^ cells upon treatment with metformin, ruxolitinib, or the combination. Particularly, SET2 cells showed lower levels of RB phosphorylation and more intense cell cycle arrest in the G_0_/G_1_ phase. Further studies are needed to determine whether other cyclin D isoforms (e.g.*, CCND2*, *CCND3*), or even small amounts of *CCND1* are sufficient to induce cell cycle progression in HEL cells. Indeed, differences in the response to metformin and/or ruxolitinib between HEL and SET2 cells were observed. HEL cells are derived from a patient with erythroleukemia^45^, featuring faster growth in culture conditions and less sensitivity to ruxolitinib, even at doses that are able to inhibit the JAK2/STAT activation. SET2 cells are derived from a patient with leukemic transformation of ET and shows slower growth, better response to ruxolitinib, and requirement for high fetal bovine serum (FBS) concentration for growth^46^. These differences among HEL and SET2 cell lines may affect cell response to metformin.

Metformin has been described as a mitochondrial complex I inhibitor^20,47^, which has been implicated as an important mechanism involved in its antineoplastic effects on hematological malignancies^13,26^. Mitochondria play a key role in the activation of oncogenic signaling pathways as the effector organelle for cellular energy generation (adenosine triphosphate, ATP) by coupling the tricarboxylic acid cycle with oxidative phosphorylation^48,49^. Our findings indicate that metformin-mediated reduction of mitochondrial activity contributes to decreased cell viability in MPN cells, highlighting the importance of increased energy demand for the proliferation of cancer cells^50^.

Metformin treatment at a dose of 125 mg/kg/day effectively reduced tumor burden in Ba/F3 JAK2^V617F^ allograft and splenomegaly in Jak2^V617F^ knockin-induced MPN murine models. In humans, this dose is equivalent to 10.4 mg/kg/day according to the coefficient of conversion proposed by Freireich et al.^51^, which represents a pharmacological dose lower than that used for type II diabetes treatment. Metformin also reduced spontaneous erythroid colony formation in primary cells from patients with PV. Additionally, although metformin and/or ruxolitinib treatment had modest effects on the induction of apoptosis and/or cell cycle arrest in JAK2^V617F^ cell lines, prolonged exposure to these drugs (in monotherapy or in combination), in the absence of growth factors, strongly reduced autonomous growth, which is a characteristic that is usually associated with aggressiveness in leukemia cells^52,53^. Using U937 cells, we also confirm that metformin reduces cell viability in JAK2^WT^ cells (Supplementary Figure 6), corroborating the multitarget potential of metformin in hematological malignancies^9^. Based on our in vitro studies, we speculate that the current clinically indicated dose of metformin may provide better improved clinical results for such patients.

In summary, metformin exerts an antileukemic activity and downregulates JAK2/STAT signaling in JAK2^V617F^ cells. PCR-array identified cyclin D1 and p27 as contributors to the mechanism of metformin and ruxolitinib efficacy, corroborating cell cycle and proliferation findings. Our exploratory study establishes novel molecular mechanisms of metformin action alone and in combinations with ruxolitinib on JAK2^V617F^ aberrant signaling and provides insights for development of alternative/complementary therapeutic strategies for MPN.

## Materials and methods

### Cell culture and inhibitors

HEL and U937 cells were obtained from ATCC (Philadelphia, PA, USA) and SET2 cells were kindly provided by Prof. Dr. Fabíola Attié de Castro (School of Pharmaceutical Sciences of Ribeirão Preto, University of São Paulo, Ribeirão Preto, Brazil). HEL and SET2 cells harboring JAK2^V617F^ mutation, and U937 cells (JAK2 wild-type) were tested and authenticated by Short Tandem Repeat (STR) matching analysis using the PowerPlex^®^ 16 HS system (Promega, Madison, WI, USA) and the ABI 3500 Sequence Detector System (Life Technologies, Foster City, CA, USA). Ba/F3 cells expressing murine erythropoietin receptor and JAK2 wild-type or JAK2^V617F^ (named as Ba/F3 JAK2^WT^ and Ba/F3 JAK2^V617F^, respectively), and Wehi-3B cells were kindly provided by Prof. Dr. Susumu Kobayashi (Division of Hematology/Oncology, Beth Israel Deaconess Medical Center, Harvard Medical School, Boston, MA, USA). Cell culture conditions were performed in accordance with the recommendations of ATCC and DSMZ. All cell lines were mycoplasma free. Ruxolitinib was obtained from Novartis Pharmaceuticals (Basel, Switzerland). Metformin was obtained from Sigma-Aldrich (St. Louis, MO, USA).

### Cell viability assay

Cell viability was measured by MTT assay. HEL or U937 (2 × 10^4^ cells/well) and SET2 cells (4 × 10^4^ cells/well) were cultured in a 96-well plate in RPMI medium containing 10 or 20% FBS, respectively, in the presence of ruxolitinib (300 nM) and/or metformin (5 and 10 mM) for 48 h. For time-response and dose-response curves, HEL and SET2 cells were cultured as described above in 96-well plates in the presence of graded concentrations of metformin (0, 2.5, 5, 7.5, 10, 15 and 20 mM) for 24, 48 and 72 h. IC_50_ values were calculated using a nonlinear regression analysis on GraphPad Prism 5 (GraphPad Software, Inc., San Diego, CA, USA). For experiments involving Ba/F3 JAK2^WT^ or Ba/F3 JAK2^V617F^ cell lines, 2 × 10^4^ cells per well were cultured in RPMI medium supplemented with 10% FBS in a 96-well plate with or without Wehi-3B-conditioned medium, ruxolitinib (300 nM) and/or metformin (5 and 10 mM) for 48 h. At the end of each culture period, 10 μL of a 5 mg/mL solution of MTT was added to each well followed by incubation at 37 °C for 4 h. The reaction was stopped using 100 μL of 0.1 N HCl in anhydrous isopropanol. Cell viability was evaluated by measuring the absorbance at 570 nm, using an iMark™ Microplate Absorbance Reader (Bio-Rad, Richmond, CA, USA). For synergism analysis, HEL and SET2 cells were treated with graded doses of ruxolitinib (3, 10, 30,100, 300 and 1000 nM) and metformin (2.5, 5, 7.5, 10, 15 and 20 mM) alone or in combination with each other for 48 h. The CI was calculated using CompuSyn software (ComboSyn, Inc., Paramus, NJ, USA), and the data obtained were interpreted according to Chou^54^ and illustrated using multiple experiment viewer (MeV) 4.9.0 software (http://www.tm4.org/mev/).

### Apoptosis assay

HEL, SET2, Ba/F3 JAK2^WT^, Ba/F3 JAK2^V617F^ and U937 cells were seeded in 24-well plates and treated with ruxolitinib (300 nM) and/or metformin (5 or 10 mM) for 48 h. Cells were then washed twice with ice cold PBS and resuspended in binding buffer containing 1 μg/mL propidium iodide (PI) and 1 μg/mL APC-labeled annexin V. All specimens were acquired by flow cytometry (FACSCalibur; Becton Dickinson) after incubation for 15 minutes at room temperature in a light-protected area and analyzed using FlowJo software (Treestar, Inc., San Carlos, CA, USA).

### Assessment of cell proliferation by Ki-67 staining

HEL and SET2 cells were treated with ruxolitinib (300 nM) and/or metformin (5 or 10 mM) for 48 h, fixed with 70% ethanol and stored at −20 °C. Ba/F3 JAK2^V617F^ cells were treated with ruxolitinib (300 nM) and/or metformin (5 or 10 mM) for 24 h, fixed with 70% ethanol and stored at −20 °C. Ki-67 staining was performed following the manufacturer’s instructions (Ki-67 FITC clone B56; BD Bioscience, San Jose, CA, USA) and the mean of fluorescence intensity (MFI) was obtained by flow cytometry using a FACSCalibur instrument (Becton-Dickinson). IgG isotype was used as negative control for each condition.

### Cell cycle analysis

Cell cycle phases were determined using BD Cycletest™ Plus DNA Reagent Kit (Becton-Dickinson, Mountain View, CA, USA) according to the manufacturer’s instructions. In brief, HEL and SET2 cells were cultured in the presence of ruxolitinib (300 nM) and/or metformin (10 mM) for 48 h. Ba/F3 JAK2^V617F^ cells were cultured without Wehi-3B-conditioned medium, in the presence of ruxolitinib (300 nM) and/or metformin (10 mM) for 24 h. DNA content distribution was acquired in a FACSCalibur cytometer (Becton-Dickinson) and analyzed using FlowJo software (Treestar, Inc.).

### Colony formation assay

Colony formation capacity was evaluated out in semisolid methylcellulose medium (1 × 10^3^ cells/mL for HEL cells, 2.5 × 10^3^ cells/mL for SET2 cells and 1 × 10^3^ cells/mL for Ba/F3 JAK2^V617F^ cells; MethoCult 4230; StemCell Technologies Inc., Vancouver, BC, Canada). Colonies were detected after 10 days of culture by adding 1 mg/mL of MTT reagent and scored by Image J quantification software (US National Institutes of Health, Bethesda, MD, USA).

### Western blot analysis

Equal amounts of protein were used as total extracts, followed by SDS-PAGE, Western blot analysis with the indicated antibodies and imaging using the SuperSignal™ West Dura Extended Duration Substrate System (Thermo Fisher Scientific, San Jose, CA, USA) and Gel Doc XR+ system (Bio-Rad, Hercules, CA, USA) or ImageQuant LAS 4000 (GE Healthcare Life Sciences, Piscataway, NJ, USA). Antibodies against STAT3 (sc-7179), STAT5 (sc-835), p-p70S6K^T421/S424^ (sc-7984), p70S6K (sc-8418), PARP1 (sc-56197) and actin (sc-1616) were purchased from Santa Cruz Biotechnology (Santa Cruz, CA, USA). Antibodies against p-RB^S807/811^ (#9308), RB (#9309), p-STAT3^Y705^ (#9131S), p-STAT5^Y694^ (#9359S), p-AMPK^T172^ (#2535S), AMPK (#2532S), p-mTOR^S2448^ (#2971), mTOR (#2972), p-4EBP1^T70^ (#9455S), 4EBP1 (#9452S) and caspase 3 (#9665) were obtained from Cell Signaling Technology (Danvers, MA, USA). Antibodies against p-ERK1/2^T183/Y185^ (700012) and ERK1/2 (44654G) were from Life Technologies. Cropped gels retain important bands, but whole gel images are available in Supplementary Figure 7A-C. Band intensities were determined using UN-SCAN-IT gel 6.1 software (Silk Scientific; Orem, UT, USA).

### PI3K/AKT signaling pathway profile by PCR array

Total RNA from HEL cells treated with metfomin (10 mM) and/or ruxolitinib (300 nM) was obtained using TRIzol reagent (Thermo Fisher Scientific). The cDNA was synthesized from 1 µg of RNA using High-Capacity cDNA Reverse Transcription Kit (Thermo Fisher Scientific). PCR array was performed using the PI3K-AKT Signaling Pathway RT^2^ Profiler PCR Array kit (#PAHS-058A; SA Biosciences, Frederick, MD, USA) according to the manufacturer’s instructions. mRNA levels were normalized to those detected in untreated cells, and genes that presented a fold change ≥1.5-fold in any treatment were included in the heatmap using Heatmap builder software (The Ashley Lab, Stanford University, CA, USA). Amplification was performed in an ABI 7500 Sequence Detector System (Life technologies).

### Quantitative PCR

Total RNA from HEL and SET2 cells treated with metfomin (10 mM) and/or ruxolitinib (300 nM) was obtained using TRIzol reagent (Thermo Fisher Scientific). The cDNA was synthesized from 1 µg of RNA using High-Capacity cDNA Reverse Transcription Kit (Thermo Fisher Scientific). Quantitative PCR (qPCR) was performed with an ABI 7500 Sequence Detector System (Life Technologies) with specific primers for *CCND1* (Cyclin D1, forward: CTCGGTGTCCTACTTCAAATG; reverse: AGCGGTCCAGGTAGTTCAT), *CDKN1B* (cyclin-dependent kinase inhibitor 1B, also known as p27, forward: ACTCTGAGGACACGCATTTGGT; reverse: TCTGTTCTGTTGGCTCTTTTGTT) and *HPRT1* (hypoxanthine phosphoribosyltransferase 1; forward: GAACGTCTTGCTCGAGATGTGA; reverse: TCCAGCAGGTCAGCAAAGAAT). The relative quantification value was calculated using the equation 2^−ΔΔCT^. A negative “no template control” was included for each primer pair.

### Ba/F3 JAK2^V617F^ tumor formation in NSG mice

NSG (NOD.Cg-Prkdc^scid^ Il2rg^tm1Wjl^/SzJ) mice were purchased from The Jackson Laboratory (Bar Harbor, Maine, USA). Experimental groups consisted of 8–10 week-old female mice that received a 100 µL (50 µL PBS plus 50 µL matrigel) dorsal subcutaneous injection of 2 × 10^6^ Ba/F3 JAK2^V617F^ cells and daily treatment by intraperitoneal injection of vehicle (PBS, *n* = 9) or metformin (125 mg/kg, *n* = 9) for 13 days. Tumor volume (V) was obtained using the formula (*V* = *W*^2^ × *L* × 0.52), where W and L are the smaller and larger diameters, respectively. The dose of metformin at 125 mg/kg/day used for mouse experiments was based on a previous study^13^, and represents a dose equivalent to 10.4 mg/kg/day for humans^51^. All experiments were approved by the Institutional Animal Care and Use Committee of Oregon Health & Science University and the Animal Ethics Committee of the University of São Paulo, and were performed according to IACUC guidelines.

### Assessment of oxygen consumption

Viable HEL, SET2 and Ba/F3 JAK2^V617F^ cells (2 × 10^6^) were cultured alone or in the presence of metformin (10 mM) and/or ruxolitinib (300 nM) for 24 h and submitted to oxygen consumption evaluation by high-resolution respirometry assay (Oxygraph-2k, Oroboros Instruments, Innsbruck, Austria). After determination of respiratory rates supported by exogenous substrates in culture media (ROUTINE state), modulators of mitochondrial function were sequentially added: oligomycin (1 mg/mL), an ATP synthase inhibitor that establishes the non-phosphorylating respiration (LEAK state); protonophore carbonyl cyanide m-chlorophenyl hydrazone (2 μM), which determines the state of maximum capacity of the electron transport system (ETS state); and antimycin A (3 μM), a mitochondrial complex III inhibitor that establishes the residual oxygen consumption due to oxidative side reactions (rox state). The DatLab software package (Oroboros) was used for data acquisition and analysis.

### Generation of JAK2^V617F^ knockin-induced MPN mice by bone marrow transplantation

For MPN phenotype induction, 5 × 10^6^ bone marrow cells from Jak2^V617F^ knockin (Jak2^WT/V617F^) mice^55^ were transplanted into lethally irradiated Pep boy mice (B6.SJL-Ptprc^a^ Pepc^b^/BoyJ, The Jackson Laboratory). A total of 5 × 10^6^ bone marrow cells from Jak2^WT/WT^ were transplanted into lethally irradiated Pep boy mice as a experimental control group. After 4 weeks, chimerism was evaluated by CD45.1 and CD45.2 markers (Becton-Dickinson) by flow cytrometry in peripheral blood. Mice with ≥70% CD45.2 cells were randomized and treated daily by intraperitoneal injection of vehicle (PBS) or metformin (125 mg/kg) for 6 weeks. At the conclusion of the experiment, animals were harvested and subjected to analysis of spleen, bone marrow and hematological parameters. Erythroid progenitors in the spleen and bone marrow were evaluated by CD71 and Ter119 markers (Becton-Dickinson) by flow cytometry. All experiments were approved by the Animal Ethics Committee of the University of São Paulo.

### Erythropoietin-independent colony formation

Mononuclear cells were isolated from peripheral blood or bone marrow from PV patients (*n* = 5, median age 52 years [range 24–74]) by Ficoll-gradient centrifugation. A total of 2 × 10^5^ cells were plated onto methocult H4535 (supplemented with cytokines and without erythropoietin, StemCell Technologies) in the presence or not of metformin (2.5 mM) and/or ruxolitinib (50 nM)^21^. Each condition was performed in duplicate. Erythroid colonies (burst-forming units plus colony-forming units-E) were counted after 14 days.

### Statistical analysis

Statistical analyses were performed using GraphPad Prism 5 (GraphPad Software, Inc.). For comparisons, Mann–Whitney test or ANOVA test and Bonferroni post-test was used. A *p* value < 0.05 was considered as statistically significant. All pairs were analyzed and statistically significant differences are indicated.

## Electronic supplementary material

Supplementary Figure Legends

Supplementary Figure 1

Supplementary Figure 2

Supplementary Figure 3

Supplementary Figure 4

Supplementary Figure 5

Supplementary Figure 6

Supplementary Figure 7

Supplementary Table 1

## References

[1] Thoennissen NH (2010). Prevalence and prognostic impact of allelic imbalances associated with leukemic transformation of Philadelphia chromosome-negative myeloproliferative neoplasms. Blood.

[2] Pardanani A (2011). JAK inhibitor therapy for myelofibrosis: critical assessment of value and limitations. Leukemia.

[3] Harrison C (2012). JAK inhibition with ruxolitinib versus best available therapy for myelofibrosis. N. Engl. J. Med..

[4] Verstovsek S (2012). A double-blind, placebo-controlled trial of ruxolitinib for myelofibrosis. N. Engl. J. Med..

[5] Vannucchi AM (2015). Ruxolitinib versus standard therapy for the treatment of polycythemia vera. N. Engl. J. Med..

[6] Chae YK (2016). Repurposing metformin for cancer treatment: current clinical studies. Oncotarget.

[7] Pollak M (2013). Potential applications for biguanides in oncology. J. Clin. Invest..

[8] Leclerc GM, Leclerc GJ, Kuznetsov JN, DeSalvo J, Barredo JC (2013). Metformin induces apoptosis through AMPK-dependent inhibition of UPR signaling in ALL lymphoblasts. PLoS. One..

[9] Scotland S (2013). Mitochondrial energetic and AKT status mediate metabolic effects and apoptosis of metformin in human leukemic cells. Leukemia.

[10] Rosilio C, Ben-Sahra I, Bost F, Peyron JF (2014). Metformin: a metabolic disruptor and anti-diabetic drug to target human leukemia. Cancer Lett..

[11] Zhang HH, Guo XL (2016). Combinational strategies of metformin and chemotherapy in cancers. Cancer Chemother. Pharmacol..

[12] Green AS (2010). The LKB1/AMPK signaling pathway has tumor suppressor activity in acute myeloid leukemia through the repression of mTOR-dependent oncogenic mRNA translation. Blood.

[13] Dalva-Aydemir S (2015). Targeting the metabolic plasticity of multiple myeloma with FDA-approved ritonavir and metformin. Clin. Cancer Res..

[14] Gwak H, Kim Y, An H, Dhanasekaran DN, Song YS (2017). Metformin induces degradation of cyclin D1 via AMPK/GSK3beta axis in ovarian cancer. Mol. Carcinog..

[15] Shi WY (2012). Therapeutic metformin/AMPK activation blocked lymphoma cell growth via inhibition of mTOR pathway and induction of autophagy. Cell Death Dis..

[16] Grimaldi C (2012). AMP-dependent kinase/mammalian target of rapamycin complex 1 signaling in T-cell acute lymphoblastic leukemia: therapeutic implications. Leukemia.

[17] Vakana E, Altman JK, Glaser H, Donato NJ, Platanias LC (2011). Antileukemic effects of AMPK activators on BCR-ABL-expressing cells. Blood.

[18] Giacinti C, Giordano A (2006). RB and cell cycle progression. Oncogene.

[19] Warmuth M, Kim S, Gu XJ, Xia G, Adrian F (2007). Ba/F3 cells and their use in kinase drug discovery. Curr. Opin. Oncol..

[20] Wheaton WW (2014). Metformin inhibits mitochondrial complex I of cancer cells to reduce tumorigenesis. Elife.

[21] Mazzacurati L (2015). The PIM inhibitor AZD1208 synergizes with ruxolitinib to induce apoptosis of ruxolitinib sensitive and resistant JAK2-V617F-driven cells and inhibit colony formation of primary MPN cells. Oncotarget.

[22] Quintas-Cardama A (2010). Preclinical characterization of the selective JAK1/2 inhibitor INCB018424: therapeutic implications for the treatment of myeloproliferative neoplasms. Blood.

[23] Taubes G (2012). Cancer research. Cancer prevention with a diabetes pill?. Science.

[24] Shi R (2015). The antileukemia effect of metformin in the Philadelphia chromosome-positive leukemia cell line and patient primary leukemia cell. Anticancer Drugs.

[25] Wang F (2015). Metformin synergistically sensitizes FLT3-ITD-positive acute myeloid leukemia to sorafenib by promoting mTOR-mediated apoptosis and autophagy. Leuk. Res..

[26] Velez J (2016). Biguanides sensitize leukemia cells to ABT-737-induced apoptosis by inhibiting mitochondrial electron transport. Oncotarget.

[27] Rosilio C (2013). The metabolic perturbators metformin, phenformin and AICAR interfere with the growth and survival of murine PTEN-deficient T cell lymphomas and human T-ALL/T-LL cancer cells. Cancer Lett..

[28] Rodriguez-Lirio A (2015). Metformin induces cell cycle arrest and apoptosis in drug-resistant leukemia cells. Leuk. Res. Treat..

[29] Bruno S (2015). Metformin inhibits cell cycle progression of B-cell chronic lymphocytic leukemia cells. Oncotarget.

[30] Voltan R (2016). Metformin combined with sodium dichloroacetate promotes B leukemic cell death by suppressing anti-apoptotic protein Mcl-1. Oncotarget.

[31] Deng XS (2012). Metformin targets Stat3 to inhibit cell growth and induce apoptosis in triple-negative breast cancers. Cell Cycle.

[32] Feng Y (2014). Metformin promotes autophagy and apoptosis in esophageal squamous cell carcinoma by downregulating Stat3 signaling. Cell Death Dis..

[33] Li L (2014). Metformin sensitizes EGFR-TKI-resistant human lung cancer cells in vitro and in vivo through inhibition of IL-6 signaling and EMT reversal. Clin. Cancer Res..

[34] Lin CC (2013). Metformin enhances cisplatin cytotoxicity by suppressing signal transducer and activator of transcription-3 activity independently of the liver kinase B1-AMP-activated protein kinase pathway. Am. J. Respir. Cell Mol. Biol..

[35] Zhao Z (2014). Metformin inhibits the IL-6-induced epithelial-mesenchymal transition and lung adenocarcinoma growth and metastasis. PLoS One.

[36] Pan Q (2015). Metformin can block precancerous progression to invasive tumors of bladder through inhibiting STAT3-mediated signaling pathways. J. Exp. Clin. Cancer Res..

[37] Liu Q (2016). Metformin represses bladder cancer progression by inhibiting stem cell repopulation via COX2/PGE2/STAT3 axis. Oncotarget.

[38] Yue W (2015). Metformin combined with aspirin significantly inhibit pancreatic cancer cell growth in vitro and in vivo by suppressing anti-apoptotic proteins Mcl-1 and Bcl-2. Oncotarget.

[39] Kawashima I, Kirito K (2016). Metformin inhibits JAK2V617F activity in MPN cells by activating AMPK and PP2A complexes containing the B56alpha subunit. Exp. Hematol..

[40] Walz C (2006). Activated Jak2 with the V617F point mutation promotes G1/S phase transition. J. Biol. Chem..

[41] Frank DA (2007). STAT3 as a central mediator of neoplastic cellular transformation. Cancer Lett..

[42] Matsumura I (1999). Transcriptional regulation of the cyclin D1 promoter by STAT5: its involvement in cytokine-dependent growth of hematopoietic cells. EMBO J..

[43] Fabris L (2015). p27kip1 controls H-Ras/MAPK activation and cell cycle entry via modulation of MT stability. Proc. Natl. Acad. Sci. USA.

[44] Baldassarre G, Belletti B (2016). Meet me in the cytoplasm: A role forp27(Kip1) in the control of H-Ras. Small GTPases.

[45] Martin P, Papayannopoulou T (1982). HEL cells: a new human erythroleukemia cell line with spontaneous and induced globin expression. Science.

[46] Uozumi K (2000). Establishment and characterization of a new human megakaryoblastic cell line (SET-2) that spontaneously matures to megakaryocytes and produces platelet-like particles. Leukemia.

[47] Owen MR, Doran E, Halestrap AP (2000). Evidence that metformin exerts its anti-diabetic effects through inhibition of complex 1 of the mitochondrial respiratory chain. Biochem. J..

[48] Solaini G, Sgarbi G, Baracca A (2011). Oxidative phosphorylation in cancer cells. Biochim. Biophys. Acta.

[49] Cheng Z, Tseng Y, White MF (2010). Insulin signaling meets mitochondria in metabolism. Trends Endocrinol. Metab..

[50] Wallace DC (2012). Mitochondria and cancer. Nat. Rev. Cancer.

[51] Freireich EJ, Gehan EA, Rall DP, Schmidt LH, Skipper HE (1966). Quantitative comparison of toxicity of anticancer agents in mouse, rat, hamster, dog, monkey, and man. Cancer Chemother. Rep..

[52] Lowenberg B, van Putten WL, Touw IP, Delwel R, Santini V (1993). Autonomous proliferation of leukemic cells in vitro as a determinant of prognosis in adult acute myeloid leukemia. N. Engl. J. Med..

[53] Yan Y (2009). Autonomous growth potential of leukemia blast cells is associated with poor prognosis in human acute leukemias. J. Hematol. Oncol..

[54] Chou TC (2006). Theoretical basis, experimental design, and computerized simulation of synergism and antagonism in drug combination studies. Pharmacol. Rev..

[55] Mullally A (2010). Physiological Jak2V617F expression causes a lethal myeloproliferative neoplasm with differential effects on hematopoietic stem and progenitor cells. Cancer Cell.

