# Supplementary material for: Metformin exerts multitarget antileukemia activity in JAK2V617F-positive myeloproliferative neoplasms
Source: Cell Death Dis. 2018 Feb 22;9(3):311. doi: 10.1038/s41419-017-0256-4 (PMC5833553; doi:10.1038/s41419-017-0256-4)
Supplement: Supplementary file 1 — Supplementary Figure Legends [file 41419_2017_256_MOESM1_ESM.doc]

**Supplementary Figure Legends**

**Supplementary Figure 1. Combination index of ruxolitinib and metformin in HEL and SET2 cells.** HEL **(A)** and SET2 **(B)** cells were treated with graded doses of ruxolitinib (3, 10, 30,100, 300 and 1000 nM) and metformin (2.5, 5, 7.5, 10, 15 and 20 mM) alone or in combination with each other for 48 hours and cell viability was measured by MTT assay. Combination index (CI) values were calculated using the mean of three experiments for each combination and CompuSyn software. The CI values are illustrated in the heatmaps.

**Supplementary Figure 2.** **Western blotting analysis protein phosphorylation and expression in HEL and SET2 cells.** Western blot analysis for protein phosphorylation and expression in total cell extracts from **(A)** HEL and **(B)** SET2 cells treated, or not, with ruxolitinib (300 nM) and/or metformin (5 and/or 10 mM); membranes were re-probed with the antibody for the detection of the respective total protein or actin, and developed with the SuperSignal™ West Dura Extended Duration Substrate system and a Gel Doc XR+ imaging system or ImageQuant LAS 4000. The bar graphs represent mean±SD of three independent experiments. **p*<0.05 for metformin- and/or ruxolitinib-treated cells *vs.* untreated cells; ANOVA test and Bonferroni post-test were performed, with all pairs analyzed and statistically significant differences indicated.

**Supplementary Figure 3. Metformin reduces cell viability and amplifies ruxolitinib-induced apoptosis in Ba/F3 JAK2^V617F^ cells.** Ba/F3 JAK2^WT^ or Ba/F3 JAK2^V617F^ were cultured with or without Wehi-3B-conditional medium as indicated. **(A)** Cell viability was determined by MTT assay in Ba/F3 JAK2^WT^ or Ba/F3 JAK2^V617F^ cells treated with the indicated concentrations of ruxolitinib and/or metformin for 48 hours and normalized to untreated Ba/F3 JAK2^WT^ cells cultured in the presence of Wehi-3B-conditioned medium (dashed line). Bar graphs represent the mean±SD of at least four independent experiments. **(B)** Apoptosis was detected by flow cytometry in Ba/F3 JAK2^WT^ or Ba/F3 JAK2^V617F^ cells treated with ruxolitinib and/or metformin for 48 hours using an annexin V/PI staining method. Bar graphs represent the mean±SD percent of annexin V-positive cells based on at least four independent experiments. The *p* values and cell lines are indicated in the graphs. **p*<0.05 for metformin- and/or ruxolitinib-treated cells *vs.* untreated cells, #*p*≤0.05 for metformin- or ruxolitinib-treated cells vs. combination treatment at the corresponding doses; ANOVA test and Bonferroni post-test, all pairs were analyzed and statistically significant differences are indicated.

**Supplementary Figure 4. Effects of metformin on tumor burden induced by allotransplantation of Ba/F3 JAK2^V617F^ cells.** These experiments represent the biological replicate of results shown in Figure 4. Images and volumes (mean ± SEM) of tumors induced by subcutaneous injection of Ba/F3 JAK2^V617F^ cells in NSG mice, treated with vehicle (PBS) (n=5) or metformin (125 mg/kg/day) (n=5). Tumor volume (V) was calculated using the formula V = W^2^ × L × 0.52, where W and L represent the smallest and largest diameters, respectively. Images of individual animal tumors are shown; ***p*<0.01, ANOVA test and Bonferroni post-test. Scale Bar: 10 mm.

**Supplementary Figure 5. Metformin is well-tolerated and does not modulate spleen size and hematological parameters in mice transplanted with Jak2^WT/WT^ cells. (A)** Experimental design is illustrated. Bone marrow cells from Jak2^WT/WT^ mice were transplanted into lethally irradiated Pep boy mice. After chimerism evaluation at 4 weeks, mice were randomized and treated daily with vehicle (n=5) or metformin (125 mg/kg) (n=5) for 6 weeks. **(B)** Spleen images and **(C)** weight of control and metformin-treated mice. Scale Bar: 10 mm. **(D)** Illustrative dot plots of erythroid progenitor analysis in the spleen. **(E)** Dispersion graphs showing the percentage of early erythroid progenitors (CD71^+^/Ter119^+^ cells) in spleen and bone marrow. **(F)** Representative histopathology H&E sections of spleen from vehicle and metformin-treated mice. Magnification of 40× (upper panel) and 100× (lower panel). **(G)** Dispersion graphs showing the hemoglobin and **(H)** hematocrit levels.

**Supplementary Figure 6. Metformin reduces cell viability in U937 cells. (A)** Cell viability was determined by MTT assay in U937 cells treated, or not, with the indicated concentrations of ruxolitinib and/or metformin for 48 hours and normalized to corresponding untreated cells. Bar graphs represent the mean±SD of at least four independent experiments. **(B)** Apoptosis was detected by flow cytometry in U937 cells treated with ruxolitinib and/or metformin for 48 hours using an annexin V/PI staining method. Bar graphs represent the mean±SD of at least four independent experiments quantifying apoptotic cell death. **(C)** Representative dot plots are shown for each condition; the upper and lower right quadrants (Q2 plus Q3) cumulatively contain the apoptotic population (annexin V+ cells). The *p* values and cell lines are indicated in the graphs. **p*<0.05 for metformin- and/or ruxolitinib-treated cells *vs.* untreated cells; ANOVA test and Bonferroni post-test, all pairs were analyzed and statistically significant differences are indicated.

**Supplementary Figure 7. Whole gel images of western blotting analysis**. Western blot analysis for protein phosphorylation and expression in total cell extracts from **(A)** HEL, **(B)** SET2 and **(C)** Ba/F3 JAK2^V617F^ cells treated, or not, with ruxolitinib (300 nM) and/or metformin (5 and/or 10 mM); membranes were reprobed with the antibody for the detection of the respective total protein or actin, and developed with the SuperSignal™ West Dura Extended Duration Substrate system and a Gel Doc XR+ imaging system or ImageQuant LAS 4000. The molecular weight of the ladder, antibodies, merged and unmerged images are indicated.
