# Supplementary figures and images for: Metformin exerts multitarget antileukemia activity in JAK2V617F-positive myeloproliferative neoplasms
Source: Cell Death Dis. 2018 Feb 22;9(3):311. doi: 10.1038/s41419-017-0256-4 (PMC5833553; doi:10.1038/s41419-017-0256-4)

Supplementary Figure 1

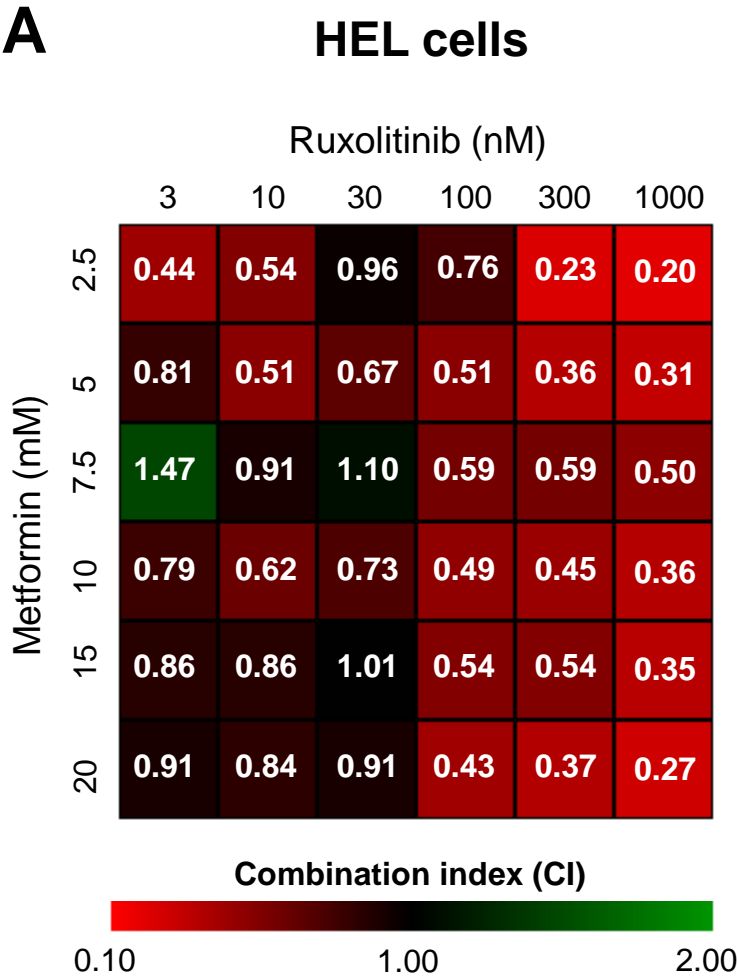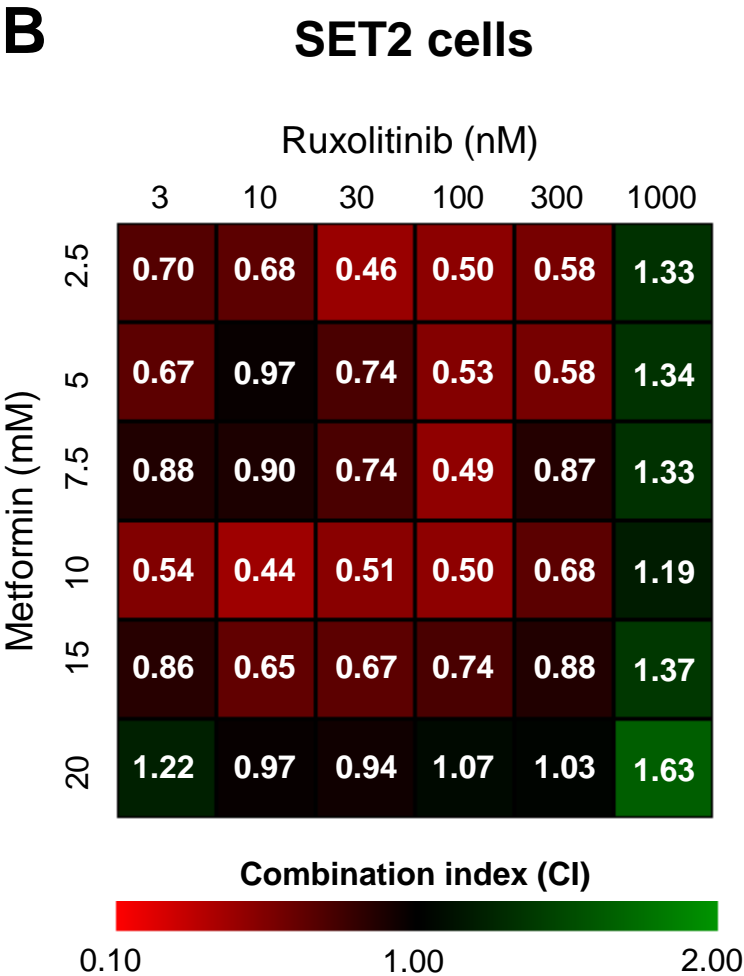

Supplement: Supplementary file 2 — Supplementary Figure 1 [file 41419_2017_256_MOESM2_ESM.pdf]

Supplementary Figure 2

**A**

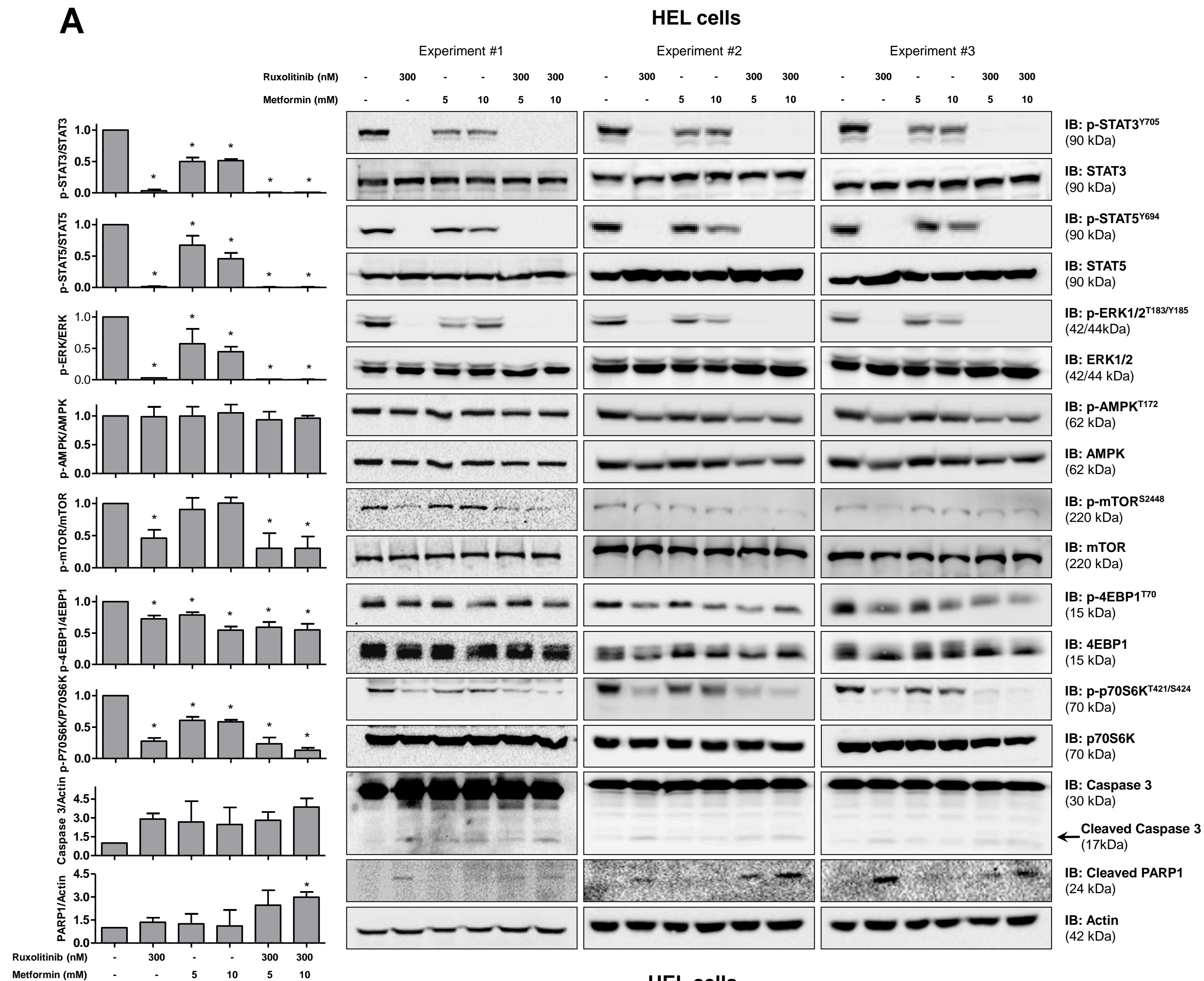

## HEL cells

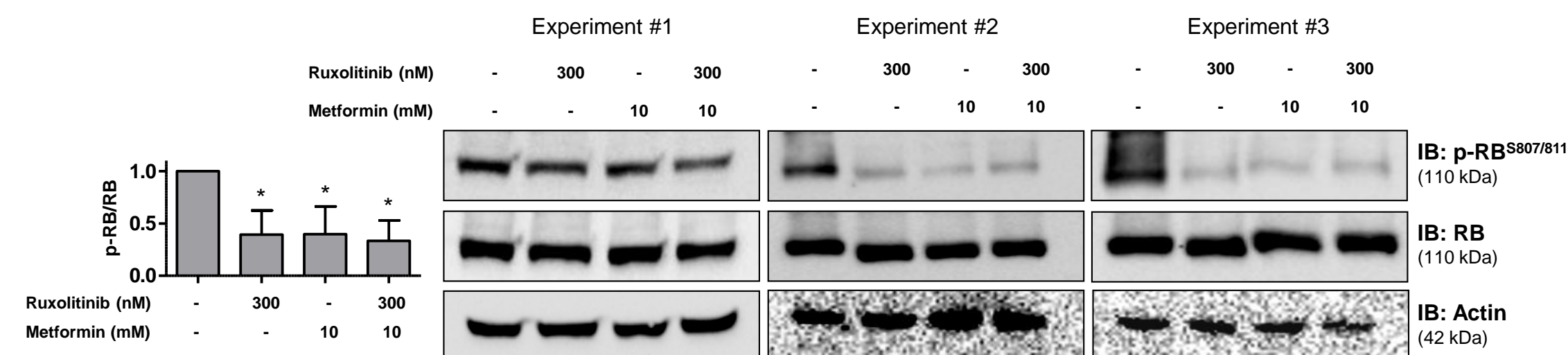

# B

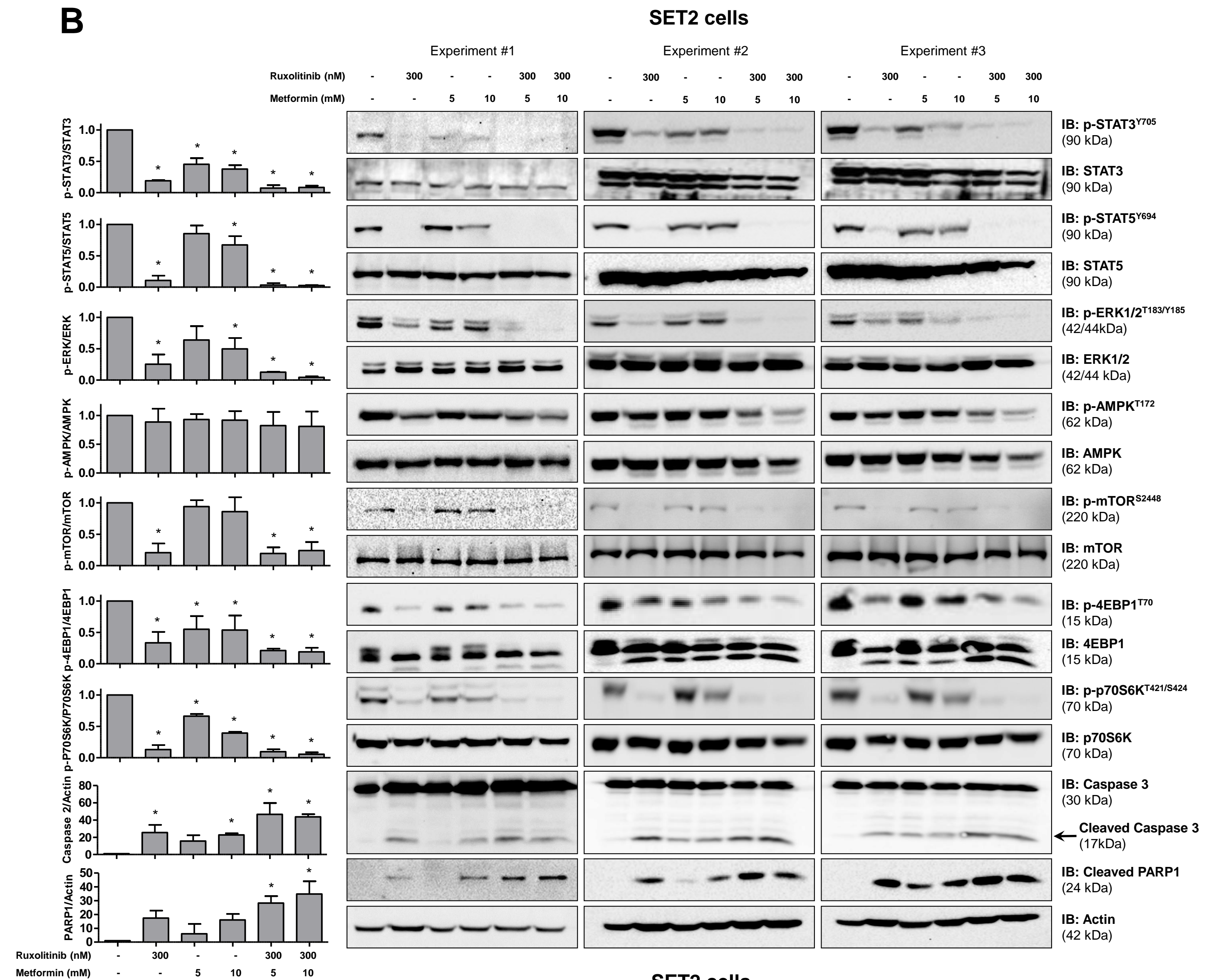

## SET2 cells

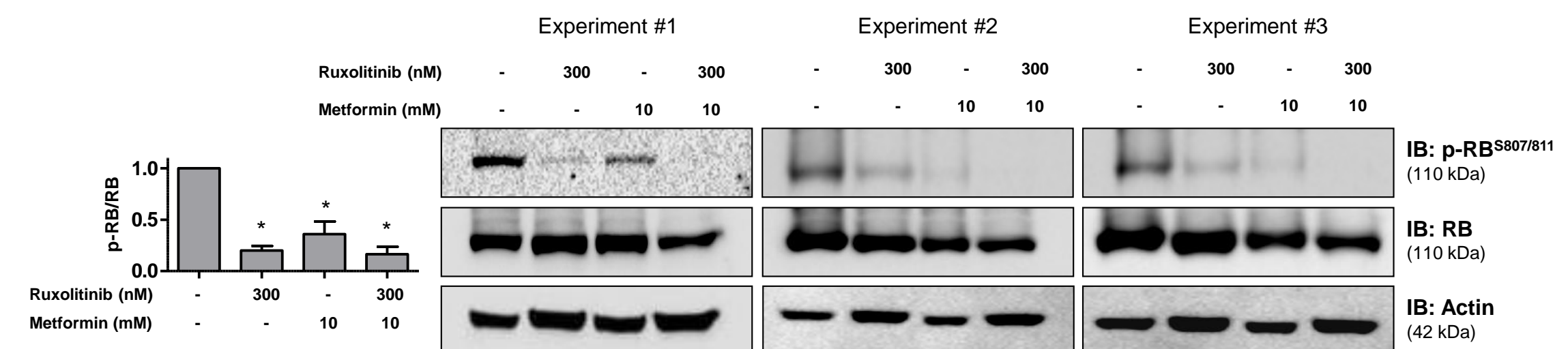

Supplement: Supplementary file 3 — Supplementary Figure 2 [file 41419_2017_256_MOESM3_ESM.pdf]

# Supplementary Figure 3

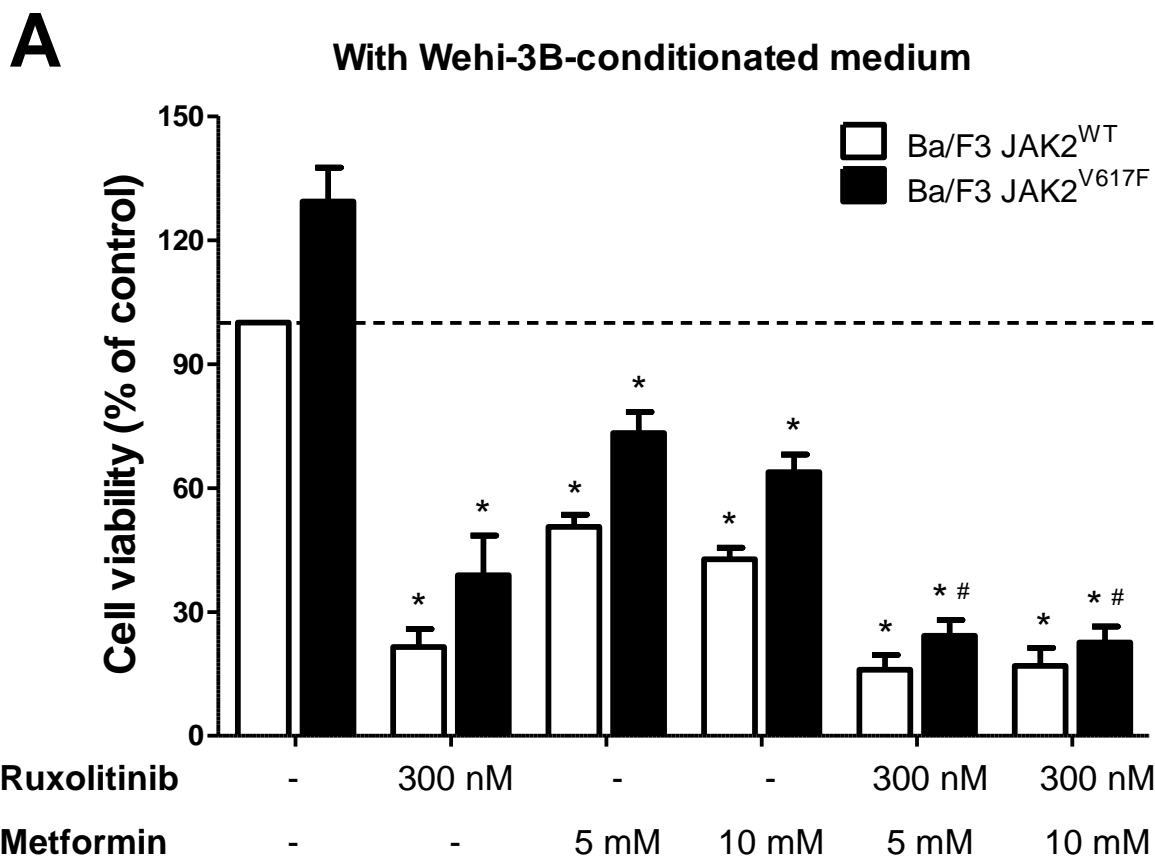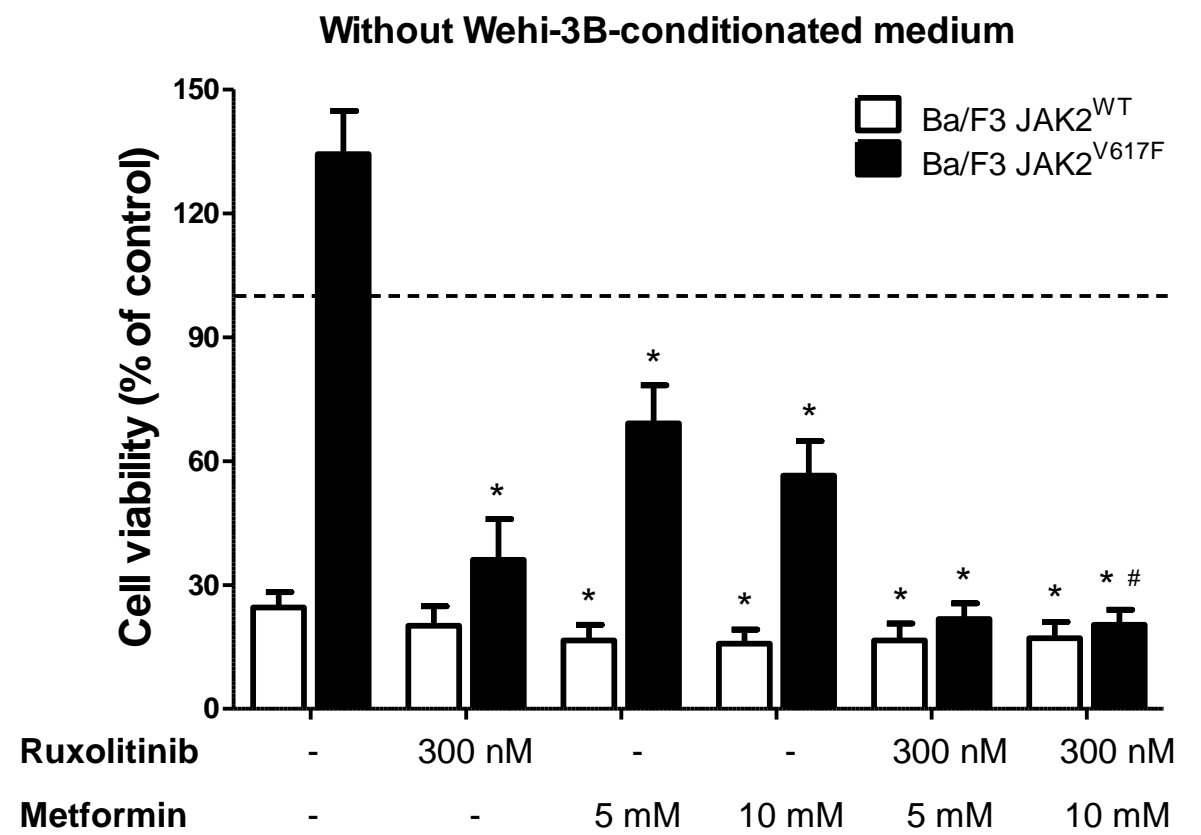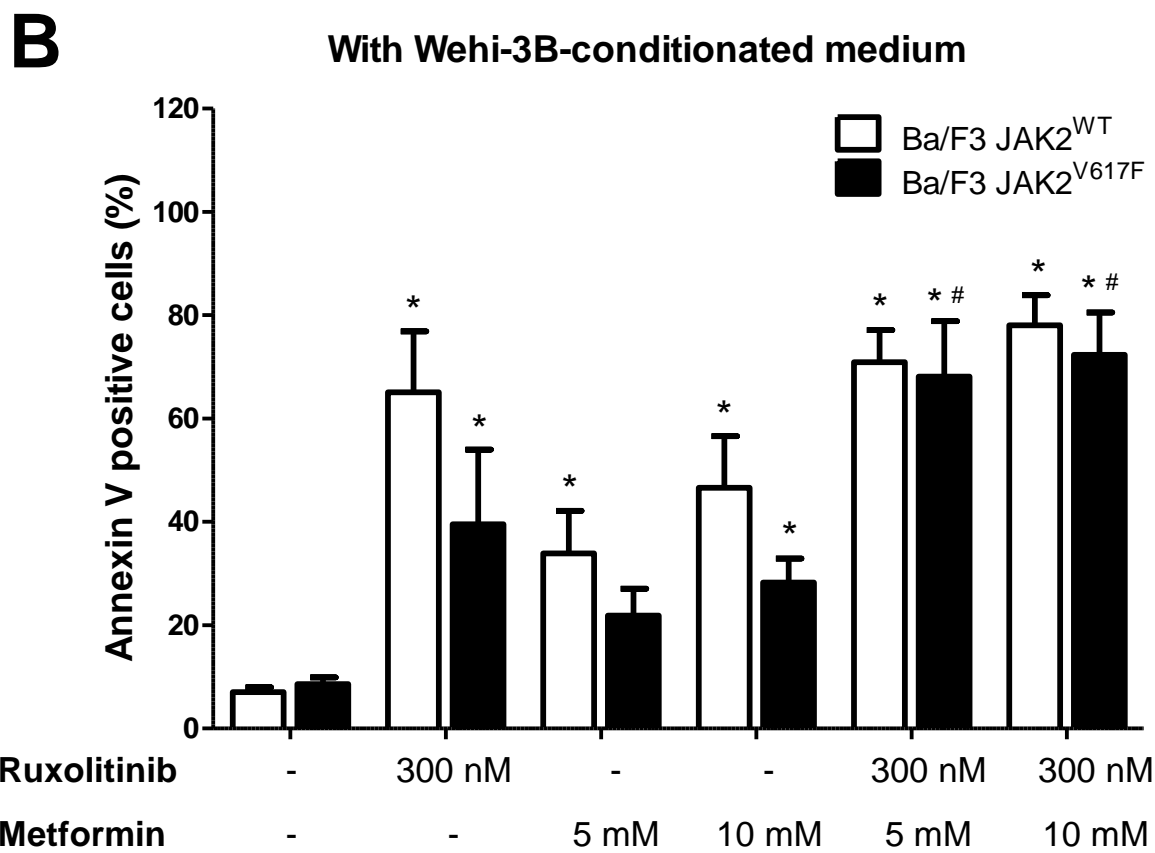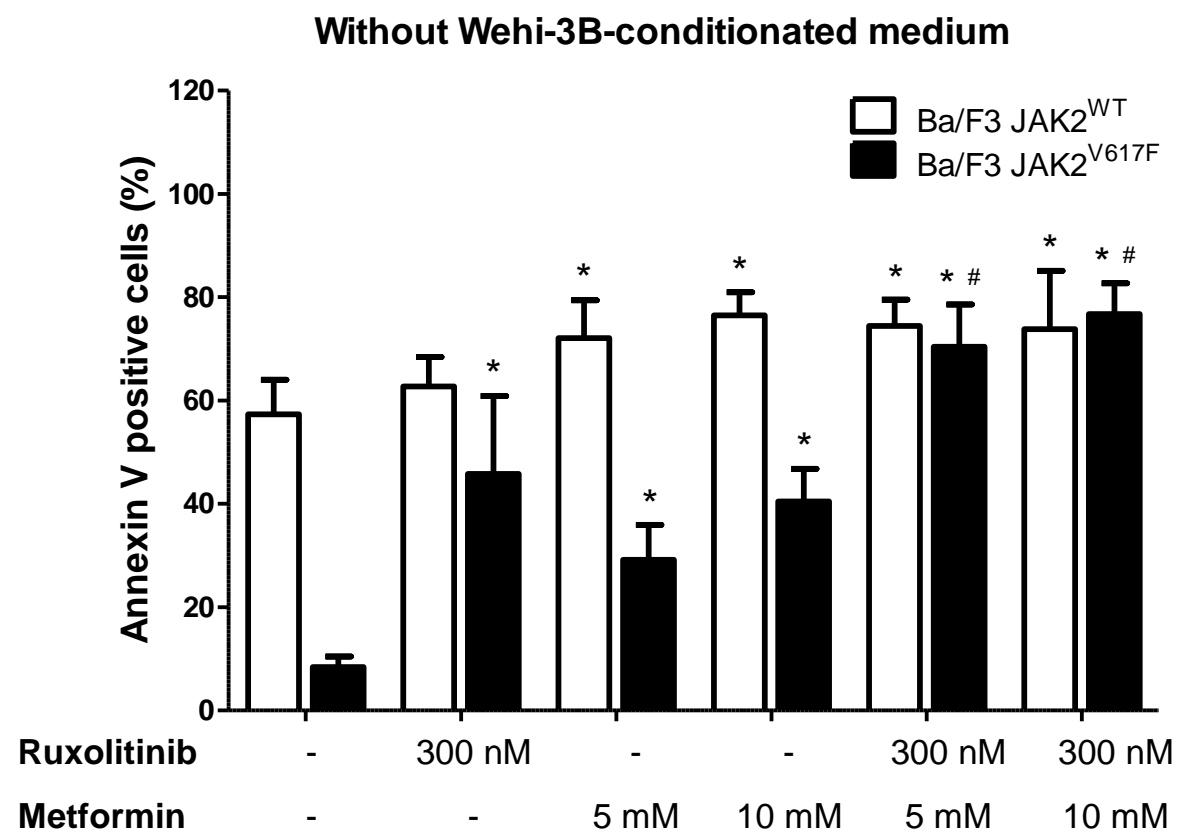

Supplement: Supplementary file 4 — Supplementary Figure 3 [file 41419_2017_256_MOESM4_ESM.pdf]

Supplementary Figure 5

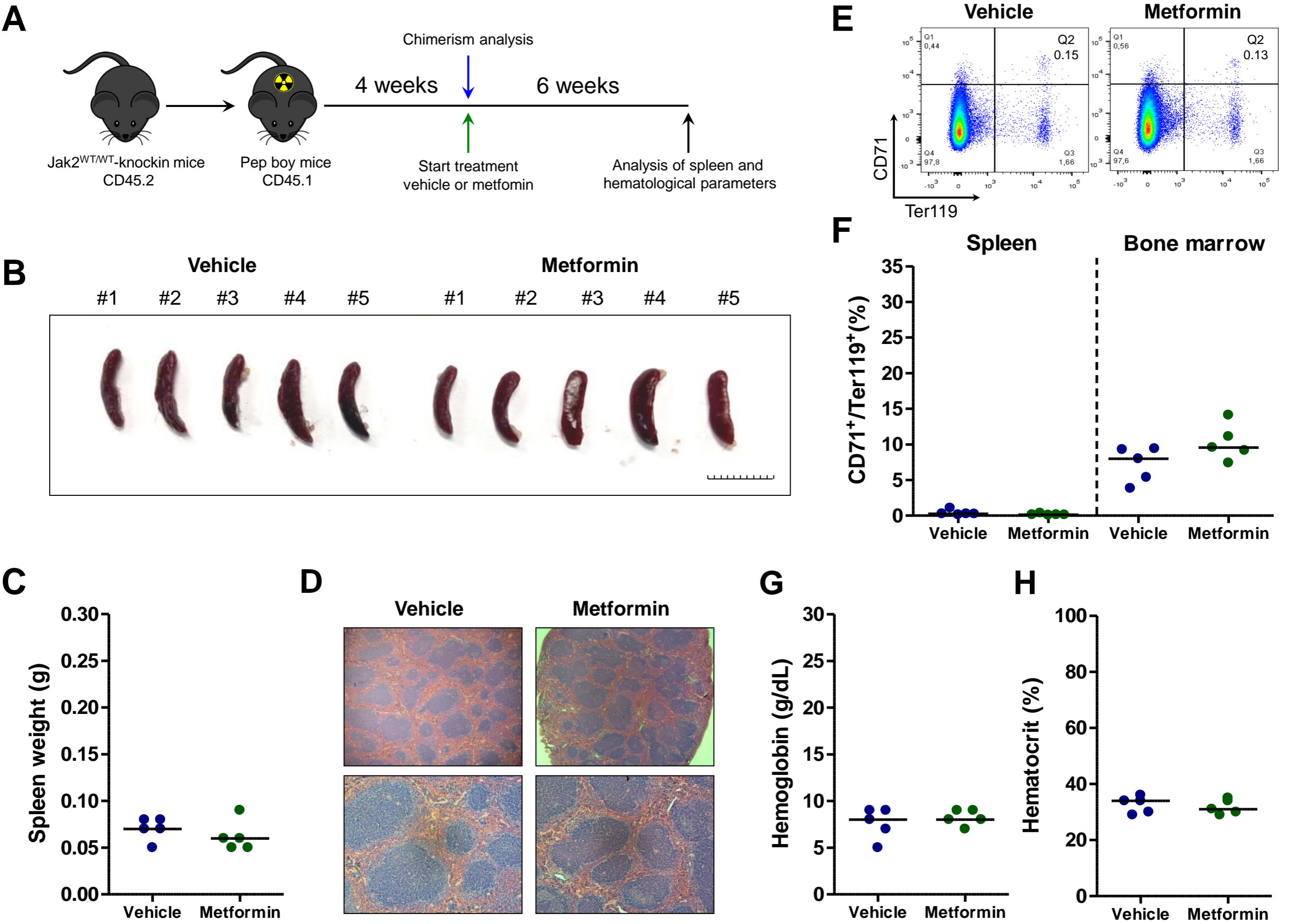

Supplement: Supplementary file 6 — Supplementary Figure 5 [file 41419_2017_256_MOESM6_ESM.pdf]

Supplementary Figure 6

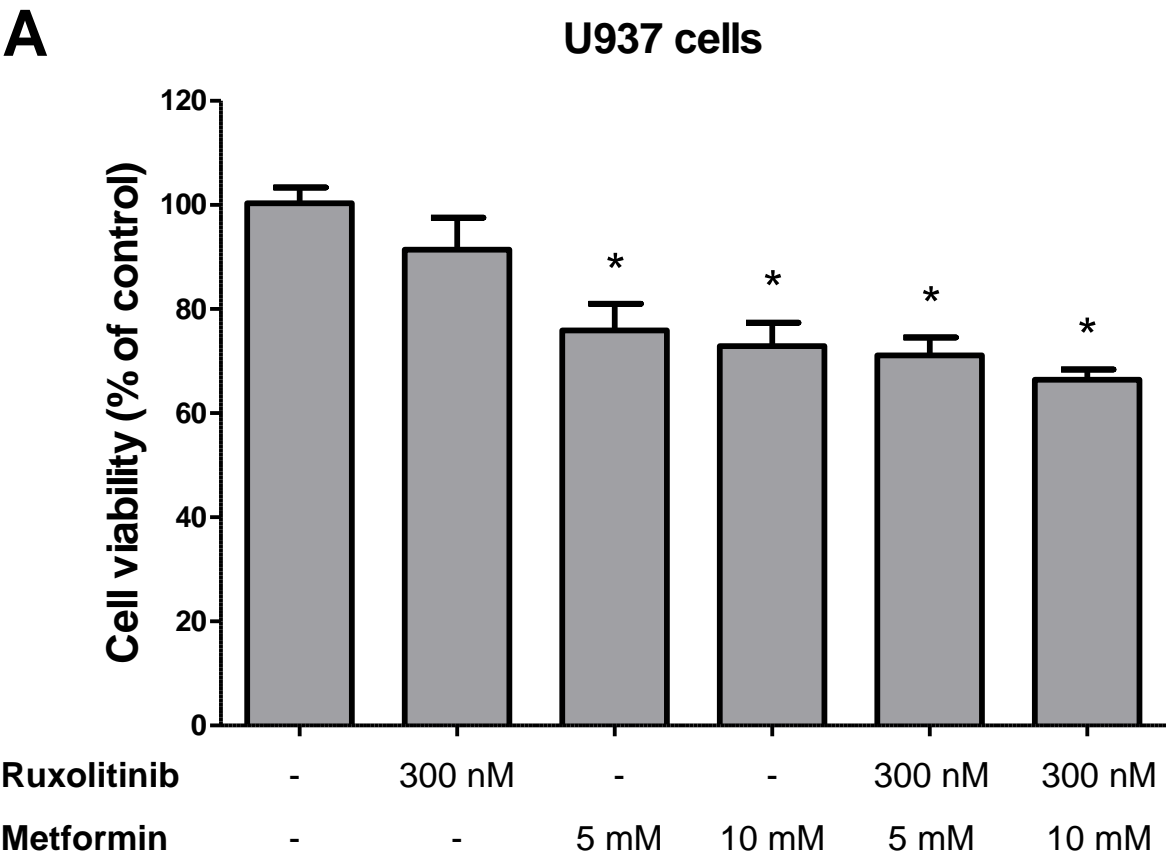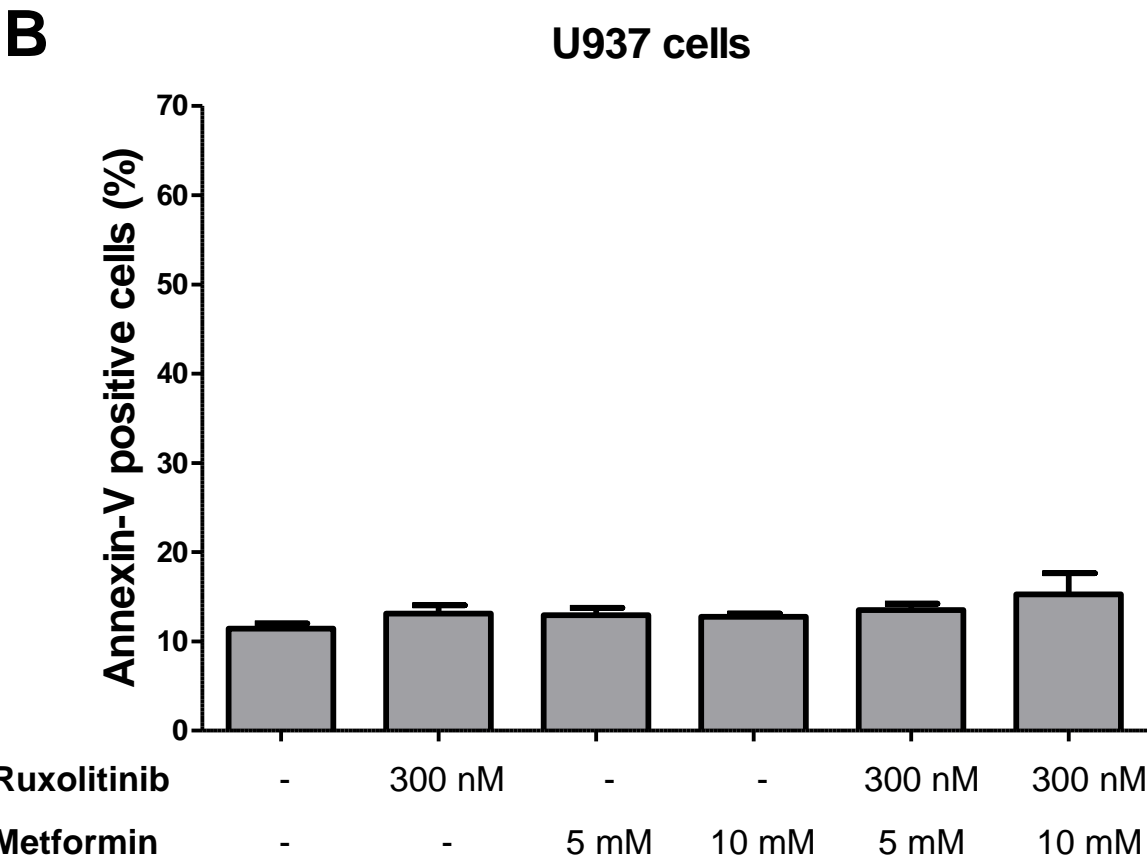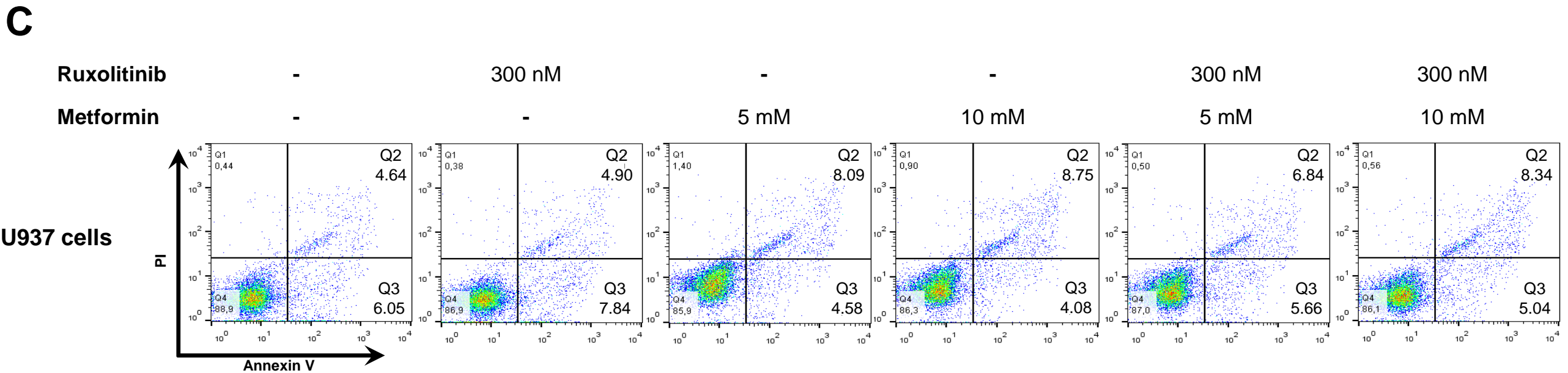

Supplement: Supplementary file 7 — Supplementary Figure 6 [file 41419_2017_256_MOESM7_ESM.pdf]

Supplementary Figure 7A

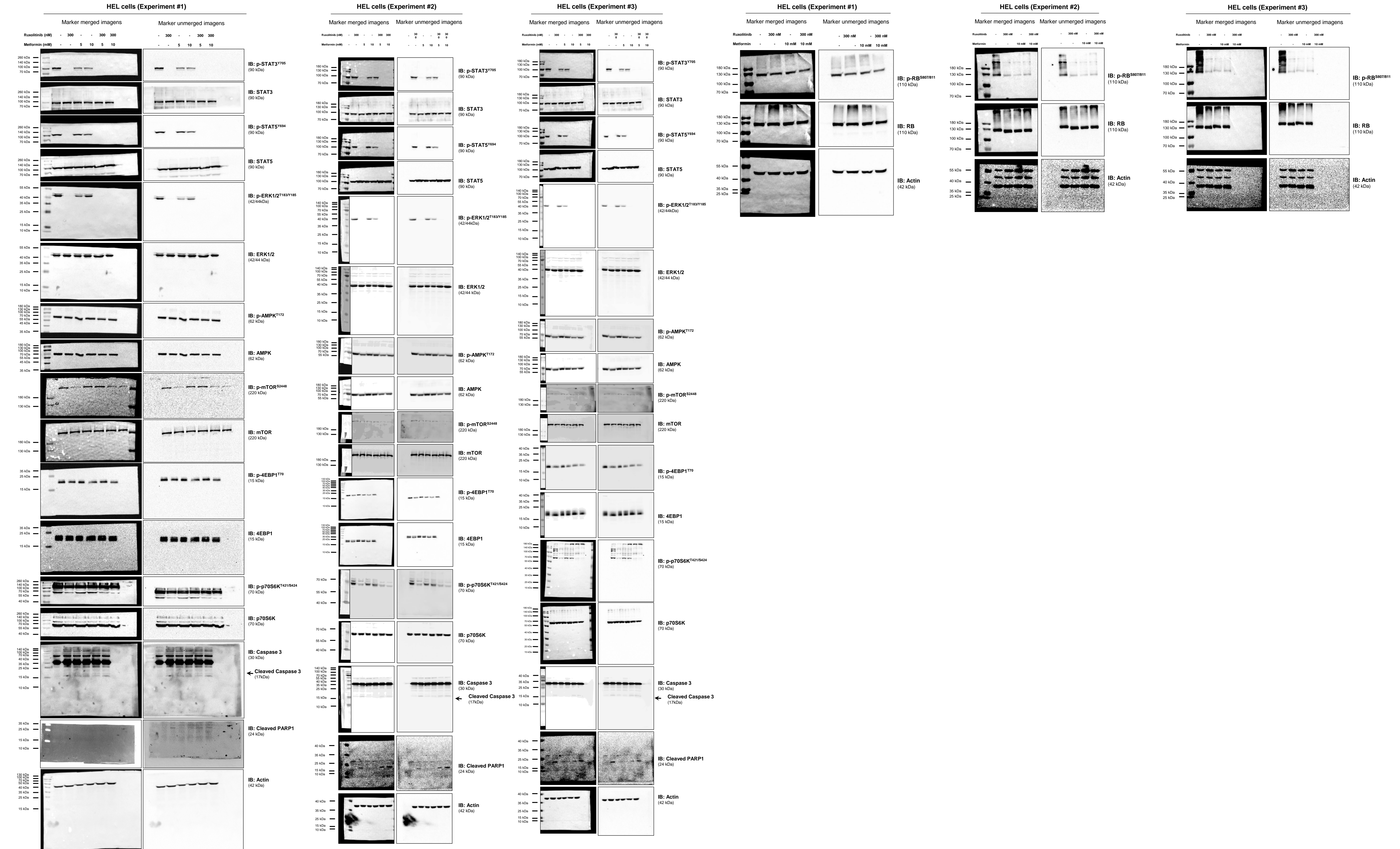

Supplementary Figure 7B

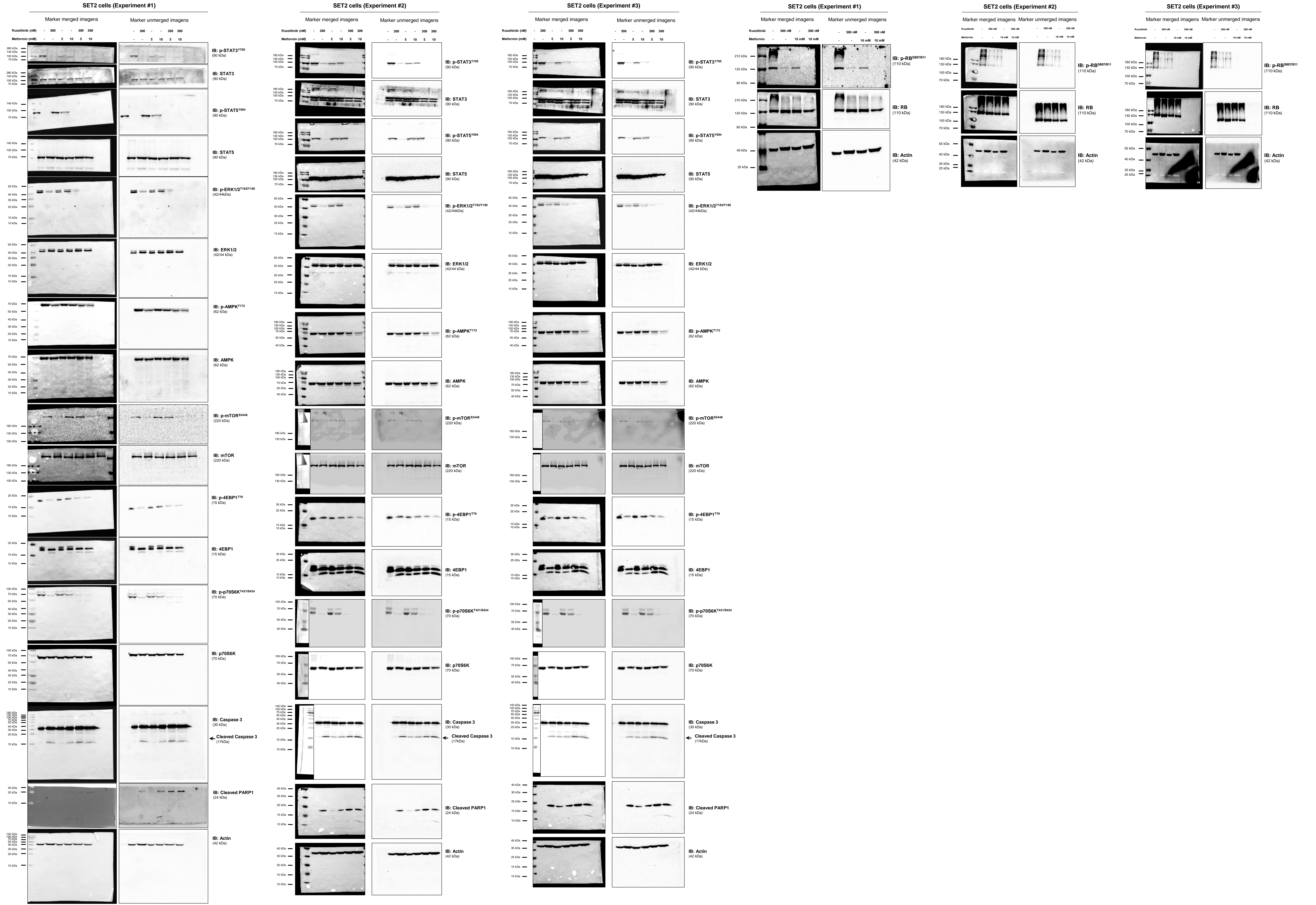

Supplementary Figure 7C

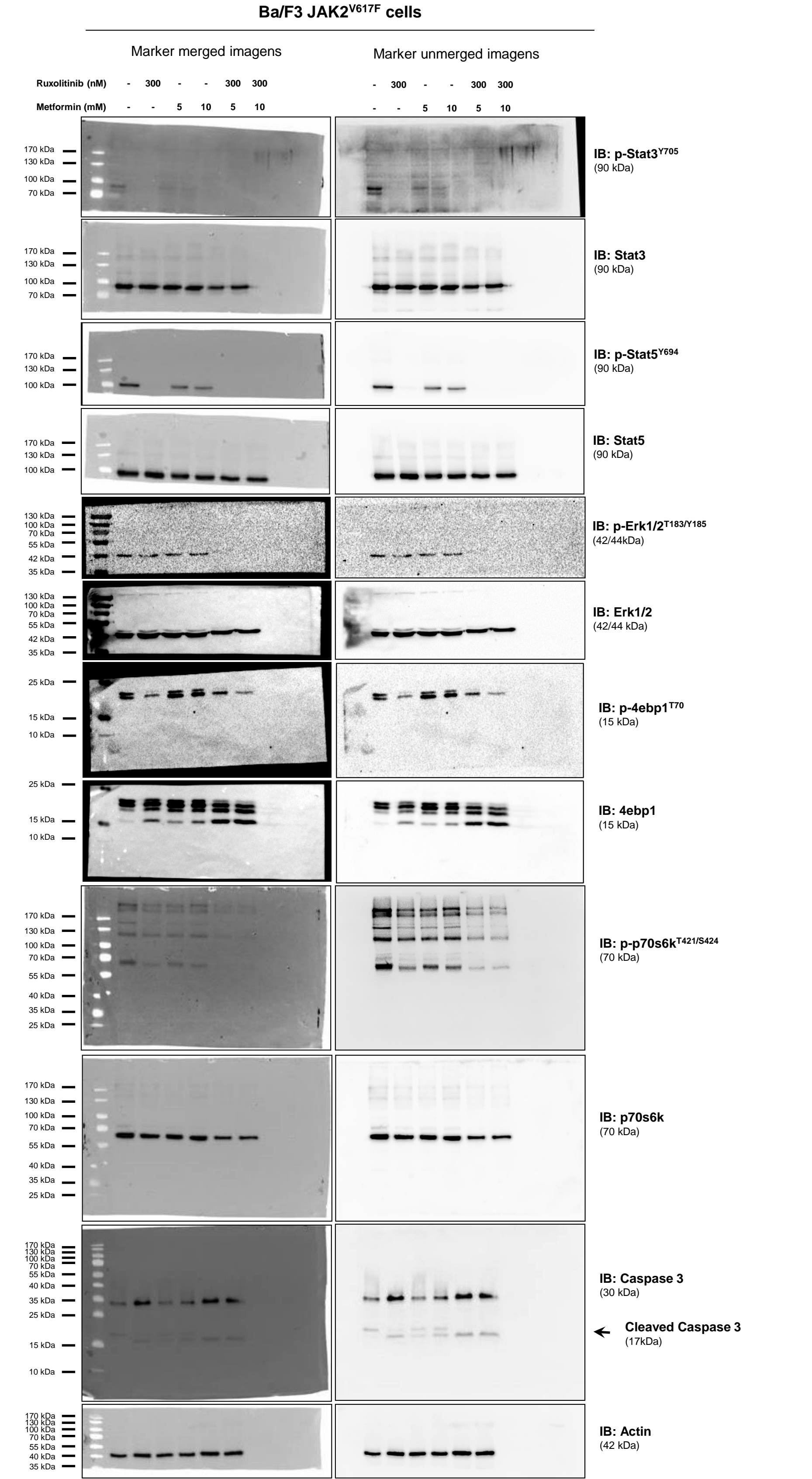

Supplement: Supplementary file 8 — Supplementary Figure 7 [file 41419_2017_256_MOESM8_ESM.pdf]
