# Supplementary material for: Metformin exerts multitarget antileukemia activity in JAK2V617F-positive myeloproliferative neoplasms
Source: Cell Death Dis. 2018 Feb 22;9(3):311. doi: 10.1038/s41419-017-0256-4 (PMC5833553; doi:10.1038/s41419-017-0256-4)

Supplementary Figure 4

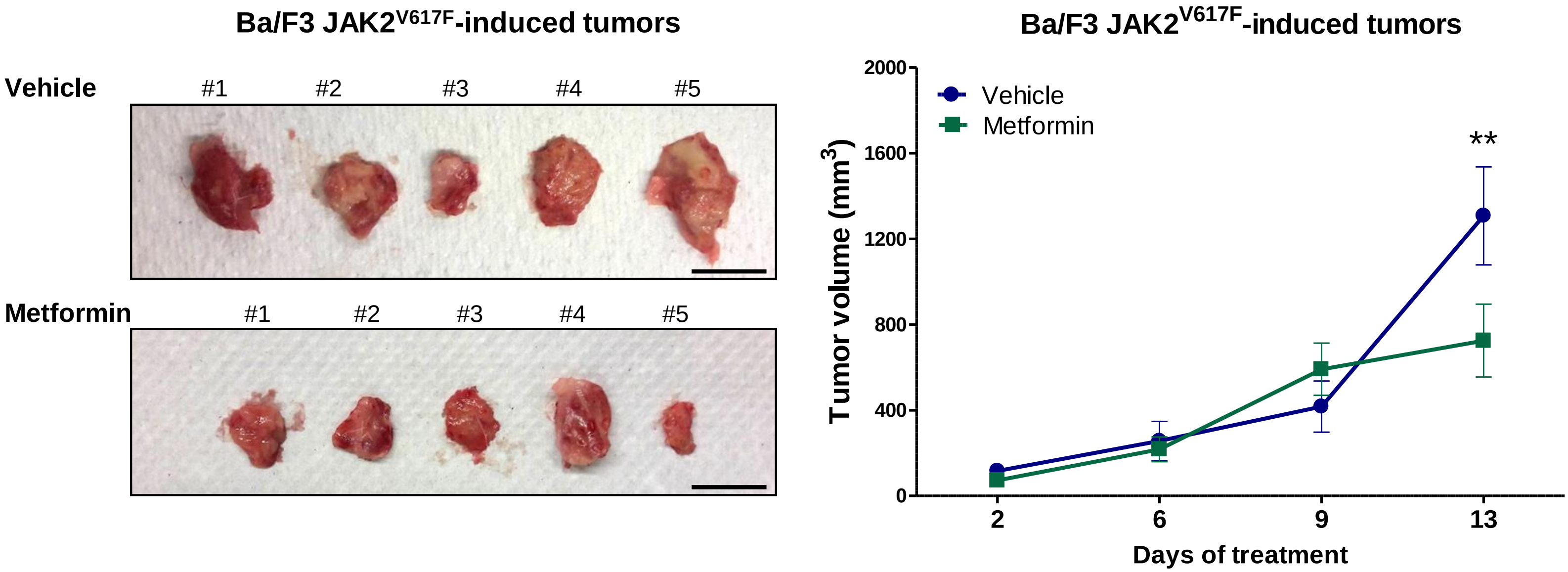

Supplement: Supplementary file 5 — Supplementary Figure 4 [file 41419_2017_256_MOESM5_ESM.pdf]
