# Supplementary material for: Metformin exerts multitarget antileukemia activity in JAK2V617F-positive myeloproliferative neoplasms
Source: Cell Death Dis. 2018 Feb 22;9(3):311. doi: 10.1038/s41419-017-0256-4 (PMC5833553; doi:10.1038/s41419-017-0256-4)
Supplement: Supplementary file 9 — Supplementary Table 1 [file 41419_2017_256_MOESM9_ESM.pdf]

Supplementary Table 1. PI3K/AKT signaling pathway-related genes investigated by PCR array

Gene	Fold change of Control		
	Ruxolitinib[1]	Metformin[1]	Ruxolitinib plus metformin[1]
ADAR	0.93	1.32	1.02
AKT1	0.94	1.06	0.98
AKT2	1.20	1.24	1.01
AKT3	1.26	**1.56**	1.29
APC	1.27	1.06	1.21
BAD	1.09	1.06	1.20
BTK	0.95	1.08	0.95
CASP9	1.17	1.14	1.31
CCND1	**0.04**	**0.33**	**0.06**
CD14	0.86	0.95	0.98
CDC42	0.81	0.89	0.75
CDKN1B	1.41	1.46	**1.55**
CHUK	0.94	1.21	1.07
CSNK2A1	0.78	0.92	0.71
CTNNB1	1.07	1.16	1.25
EIF2AK2	0.92	1.38	1.05
EIF4B	1.03	1.41	1.24
EIF4E	0.75	0.83	0.68
EIF4EBP1	0.76	1.03	0.73
EIF4G1	0.75	0.84	0.80
ELK1	0.87	0.93	0.95
FASLG	**0.39**	**0.53**	**0.23**
FKBP1A	0.77	0.89	0.79
FOS	**0.49**	0.88	**0.44**
FOXO1	**0.52**	**1.56**	1.31
FOXO3	1.19	1.37	1.33
GJA1	0.82	1.08	0.90
GRB10	**0.49**	1.01	**0.65**
GRB2	0.89	1.12	0.99
GSK3B	0.90	0.96	0.95
HRAS	1.04	1.11	1.12
HSPB1	0.86	0.80	0.81
IGF1	0.83	1.01	0.81
IGF1R	**0.62**	1.17	**0.60**
ILK	**2.39**	**1.67**	**2.72**
IRAK1	1.45	1.33	**1.51**
IRS1	1.01	1.05	1.05
ITGB1	**2.64**	**0.51**	**2.13**
JUN	0.85	1.15	0.83
MAP2K1	**0.48**	1.06	**0.57**
MAPK1	0.81	1.05	0.92
MAPK14	0.99	1.10	1.03
MAPK3	1.20	1.20	1.13
MAPK8	1.20	1.21	1.13
MTCP1	0.85	1.12	0.93

MTOR	1.14	**1.87**	**1.75**
MYD88	1.08	1.00	1.04
NFKB1	0.91	1.29	1.26
NFKBIA	0.93	1.01	1.27
PABPC1	0.95	1.20	1.06
PAK1	0.78	1.05	0.95
PDGFRA	**0.60**	**0.58**	**0.52**
PDK1	0.91	1.11	0.90
PDK2	1.40	1.17	1.18
PDPK1	1.33	1.26	1.41
PIK3CA	0.93	1.24	0.92
PIK3CG	**0.61**	0.87	**0.58**
PIK3R1	1.07	0.89	1.01
PIK3R2	1.07	1.26	1.09
PRKCA	**1.46**	**1.43**	**1.65**
PRKCB	**0.67**	1.08	**0.65**
PRKCZ	0.99	1.14	1.09
PTEN	0.96	1.15	0.99
PTK2	0.92	1.19	1.00
PTPN11	0.83	1.11	0.86
RAC1	0.82	0.89	0.82
RAF1	1.05	1.11	1.13
RASA1	1.07	1.28	1.17
RBL2	1.33	**1.53**	**1.46**
RHEB	0.84	0.96	0.79
RHOA	0.87	0.97	0.89
RPS6KA1	0.71	0.90	0.80
RPS6KB1	1.09	1.22	1.28
SHC1	0.76	0.88	0.69
SOS1	0.74	1.27	0.82
SRF	1.06	0.85	0.98
TCL1A	1.12	1.18	1.12
TIRAP	1.39	1.21	1.34
TLR4	**0.48**	**0.50**	**0.36**
TOLLIP	1.21	1.11	1.27
TSC1	0.99	1.20	1.06
TSC2	**1.51**	1.15	1.26
WASL	1.20	**1.88**	**1.99**
YWHAH	0.97	1.13	0.98

[1]Ruxolitinib and metformin were used at 300 nM and 10 mM, respectively.
Relative gene expression that presents 1.5 fold-change of control is highlighted in bold.
